# An interdisciplinary approach to Iron Age Mediterranean chronology through combined archaeological and ^14^C-radiometric evidence from Sidon, Lebanon

**DOI:** 10.1371/journal.pone.0274979

**Published:** 2023-03-09

**Authors:** Claude Doumet-Serhal, Stefanos Gimatzidis, Bernhard Weninger, Constance von Rüden, Karin Kopetzky

**Affiliations:** 1 Laboratoire UMR 8167 Orient et Méditerranée, CNRS, Paris, France; 2 Director of the Sidon Excavations, Sidon, Lebanon; 3 Austrian Archaeological Institute, Austrian Academy of Sciences, Vienna, Austria; 4 Institute of Prehistory, University Cologne, Köln, Germany; 5 Institute for Archaeological Studies - Pre- and Protohistory, Ruhr-University Bochum, Bochum, Germany; New York State Museum, UNITED STATES

## Abstract

The construction of the Iron Age Mediterranean chronology began in the Levant based on historical evidence and has been additionally supported in recent decades by means of radiocarbon analysis, although with variable precision and ratification. It is only in recent years that new evidence in the Aegean and the western Mediterranean has opened discussion towards its further acceptance as an authoritative i.e. highly reliable, and widely applicable historiographic network. Altogether, the Mediterranean Iron Age chronology has only undergone minor changes during the last hundred years. The Phoenician metropolis of Sidon in southern Lebanon now provides a new, large and robust dataset obtained through a combination of archaeological and ^14^C-radiometric analysis of materials from stratified contexts that allow their statistical assessment. The appearance of substantial amounts of pottery of Greek, Cypriot and Egyptian origin together with Phoenician local wares in a long stratigraphy is a benefit for the synchronisation of regional pottery styles and allows wider geographic correlation of relative chronological systems. The close association of the archaeological data with a long series of AMS-^14^C-dates on short-lived samples provides new evidence for the absolute dating of many of the regional pottery styles that are represented in the stratigraphy of Sidon, and contributes towards a considerable improvement of the Mediterranean chronology.

## Introduction

**Fig 1:**
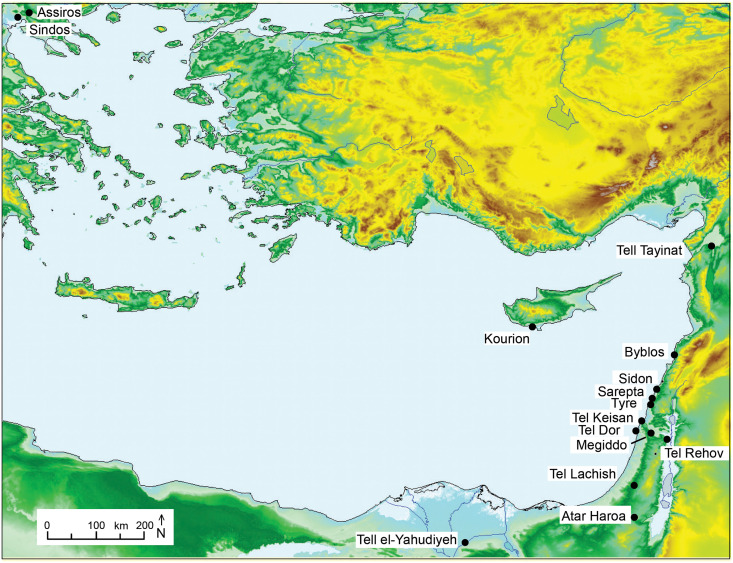
Map showing the Aegean and the eastern Mediterranean with the sites mentioned in the text (Produced by Globalmapper© based on SRTM-30 data with WGS84 projection).

Many details of the Iron Age chronology of the Mediterranean have been questioned during the last two decades by new dendro- and radiocarbon data from several sites in the Aegean, central and eastern Mediterranean as well as through archaeological critique [1–7]. Some of these studies, that were conducted in the central Mediterranean, put into question for the first time the conventional Mediterranean chronology [1–3], an otherwise vague term that has not yet been adequately defined. More recent data from the Aegean [4, 6] stressed the need for a revision of conventional Iron Age chronology based on robust stratigraphic data. In the meantime it became evident that a new definition of the Mediterranean chronology is required, one based on the systematic correlation among many different regional relative chronological systems that have been developed over the years and which range from the Iberian Peninsula to Cyprus and the Levant, and from Italy and Greece to the northern African coast. These ambitious endeavours were based primarily on relatively dated artefacts with often only assumed primary sourcing in certain microregions, and which were widely circulated throughout the Mediterranean. A favourite tool for this purpose was the Greek Early Iron Age and Archaic pottery that earned its good reputation due to its fine and well-dated sequence. Greek pottery was indeed one of the most commonly, widely used and archaeologically visible commodities in the Mediterranean, from the Atlantic coasts of Spain through to the Levantine coast as well as Mesopotamia. The absolute dates for the Greek ceramic sequence were, however, not obtained in the Aegean but in the eastern Mediterranean, where actually quite sparse Greek pottery finds have been recovered in historically, allegedly well-dated contexts. Interestingly, many of these early synchronisms that were based on evidence from culture-historical methods were later confirmed, at first sight, by scientific dating in Israel. It is this evidence that has been recently questioned [1–7] and which is further scrutinized in this paper through juxtaposition to our new ^14^C-AMS-data from the site of Sidon (Fig 1). This large radiocarbon dataset is not only valuable for the dating of regional ceramic sequences but also provides an opportunity to reevaluate the Iron Age Mediterranean chronology based on new scientific and archaeological evidence. The Early Iron Age levels at Sidon’s College Site that provided our AMS-data yielded, next to local Phoenician pottery, one of the largest assemblages of well-dated Protogeometric and mainly Geometric Greek pottery in the Levant as well as Cypriot and Egyptian wares. In this way the new scientific and archaeological data from Sidon provides us with a starting point for reexamination of the Iron Age chronology in a wider Mediterranean context. We approach this topic, in the present paper, through a study of all predominant regional pottery categories, as well as on the basis of comprehensive ^14^C-analysis of the AMS-data from Sidon and other sites.

The first and most significant impact of our dating project is on the Iron Age chronology of what is known as Phoenicia, most of which is today in modern Lebanon. A substantial amount of literature on the chronology of the southern Levant in the Late Bronze and Early Iron Age transpired already from the screening, selection, and explorative stratigraphic modelling of over 350 radiocarbon dates carried out on samples from prominent sites such as Megiddo (N = 147), Tel Rehov (N = 113), Tel Dor (N = 21), Tel Lachish (N = 61), and Ata Haroa (N = 16). Put together, for Israel, the CalPal-database contains a total of around 100 ^14^C-dates for the Late Bronze Age and more than 350 ^14^C-dates for the Iron Age. But the data availability is not only highly disperse, in terms of sampling quality and dating precision, but also in geographic terms. In contrast to Israel, on the northern littoral of Levantine Phoenicia, the tells in long settled sites such as Tyre and Sarepta with their long stratigraphic sequences dating up to the Iron Age, have up until now, only been correlated to sites in the southern Levant by means of ceramic typological criteria [8]. Phoenicia has long been considered one of the few areas where a smooth transition between the Late Bronze and Iron Age took place thus accounting for the interchangeable use of the terms ‘Canaanite’ and ‘Phoenician’ [9–11]. This smooth transition is further attested by the Early Iron Age material culture that remained infused with what is known as the Canaanite tradition [12].

Located in the heartland of Phoenicia, Sidon offers an unprecedented sequence of occupation for this transitional period. Significant evidence has emerged from Sidon relating first, to the ongoing discussion about the chronology of the Early Iron Age, shaped by a certain regionalism as deduced from the material culture and secondly to the chronological sequence between the southern and the northern Levant, resulting in a clearer picture of Phoenicia as a whole. Additionally, the city abounded with overseas Mediterranean contacts, specifically with Greece, Cyprus and Egypt, and this underpins the importance of Sidon’s chronology in its geopolitical context. Further commercial and cultural ties with Cyprus and the Aegean are embodied in the use of imported pottery from these regions. Cypriote vessels, representative of the network of exchanges included a probable Proto-White Painted vessel in Phase A together with Cypro-Geometric I/Cypro-Geometric II pottery for Phases B to E, while vessels from Cypro-Geometric II/III were found from Phase E onwards [13]. Pottery from the Aegean comprised types such as pendent semicircle plates and skyphoi that were commonly used also at several sites in the eastern Mediterranean. However, the relatively large quantity and some of the types of well-dated Greek Geometric pottery found in the excavated area of the ‘College site’ are uncommon outside of Sidon. Among them there is an exceptional find, a large Euboean crater of the so-called Cesnola Painter with the Tree of Life and two rampant goats at each side. This crater, with a parallel in a mortuary context at Kourion, Cyprus, must have reached Sidon as a special order as attested by the ritual context it was used in [14]. Commercial ties with Egypt are illustrated through the import of commodities packed in jars and pottery vessels that reached Sidon at the end of the 20^th^ beginning of the 21^st^ Dynasties [15]. The importance of the city in this time period is illustrated in the Tale of Wenamun, which narrates the journey of an Egyptian high official in temple administration who travelled to Phoenicia in the late 20th or more likely the very early 21st Dynasty and which describes the fleet anchored in Sidon’s harbour [12, 16] as being larger than the one in Byblos.

## Archaeological and analytical methods

### The architectural remains and the stratigraphical sequence at Sidon

The Sidon ‘College site’ excavation, which started in 1998 and is still ongoing, has revealed a total of twelve stratigraphic phases dating to the Iron Age, labelled as Phases A to L, with an addition of three Subphases (C1, D1 and D2) [12]. The Sidon stratigraphic sequence was reconstructed through detailed studies of the architecture and is chronologically supported by means of a large and variable ceramic assemblage as well as a substantial set of radiocarbon ages (N = 37, cf. Tab.BW-1), mainly obtained on short-lived olive pits and animal bones from floors or features associated with them, and measured to high precision by the Oxford and Mannheim AMS-Laboratories.

The stratigraphic and ceramic sequences of Sidon are unique in the region, narrating the evolution of the city in this time period. This is the first time that a site in Lebanon provides a tight chronological framework for the Early Iron Age and has thus allowed the construction of a time-related cultural framework for this period in Phoenicia.

**Fig 2:**
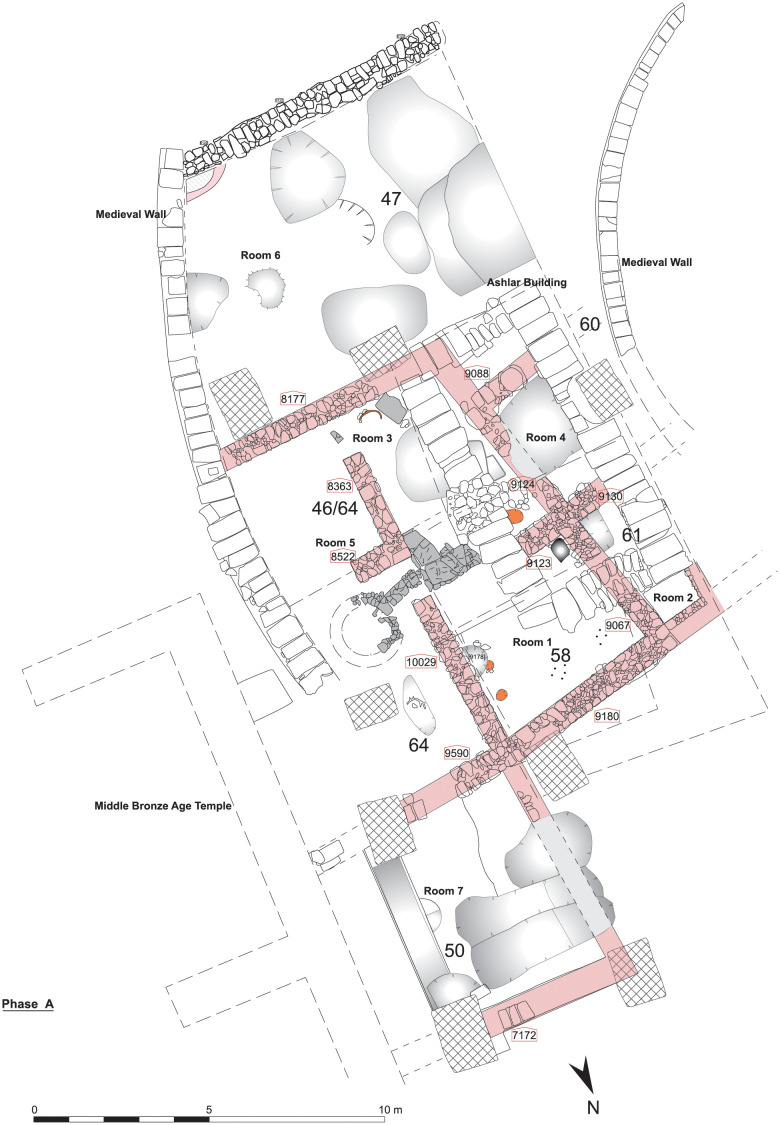
The Temple, Phase A.

**Fig 3:**
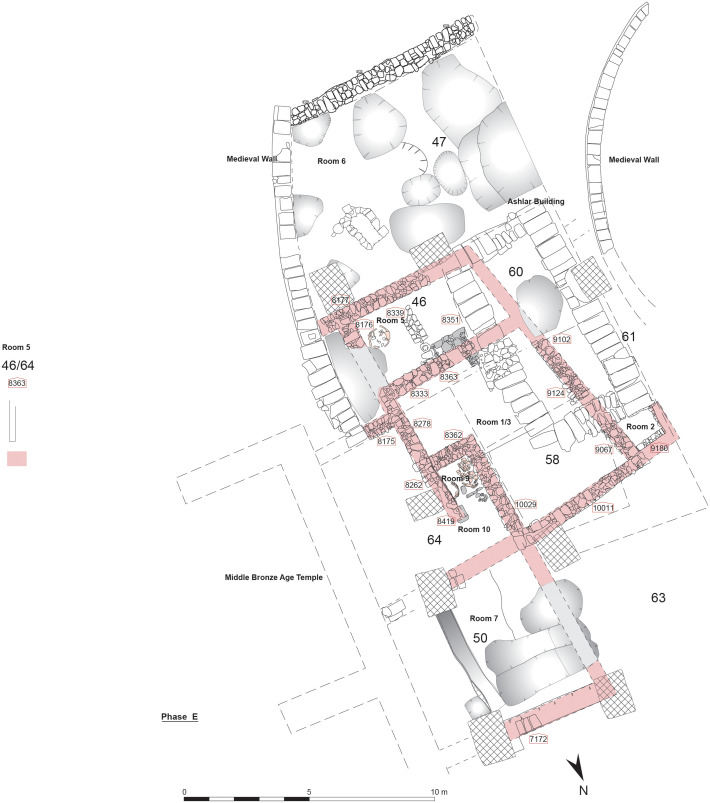
The Temple, Phase E.

Sometime during the end of the 13^th^ or the beginning of the 12^th^ century BC, a large building that must have been used for ritual purposes, was erected in Sidon (Fig 2). The 24.80 m long edifice is not well preserved due to later disturbances. However, it still represents a rare find for this time period [9]. A total of 10 rooms were excavated. Over time, the basic outer plan of this building, that must have functioned as a temple, remained largely unchanged with only the inner layout changing twice. The first alterations in the plan of the building took place in Phase E (Fig 3) and this layout was used without further changes in Phase F; Phase G marked a second major change in the building plan that was kept until Phase J. This means that four major stages occurring in the plan and space use of the building’s interior, namely Phases A to D dating mainly to the 12^th^ and 11^th^ centuries; Phases E, F, G that date to the 10^th^ century BC; Phases H, I, J, dated to the late 10^th^ and 9^th^ century, Phase K to the end of the 9^th^ beginning of the 8^th^ century BC and finally phase L. In architectural terms, the Sidon building belongs to the vernacular style of temples. There is nothing unusual about the materials used for its construction to make its special role immediately apparent. The continuity in the use of the sacred space is nevertheless attested by several finds with ritual implications that were distributed throughout its stratigraphic sequence as well as the continuous adaptation and change in the layout of the rooms to suit needs in the use of space through centuries [12]. While the boundaries between household and public ritual practice are not always straightforward [12] ritual activities in the building can be traced in its architectural furnishing with niches and benches-along-the-wall and a possible altar made of coarse stones [12] combined with the discovery of finds such as an excess of 100 astragali, special vessels such as chalices, goblets and stands, and an ivory panel featuring a standing male grasping the tree of life.

**Fig 4:**
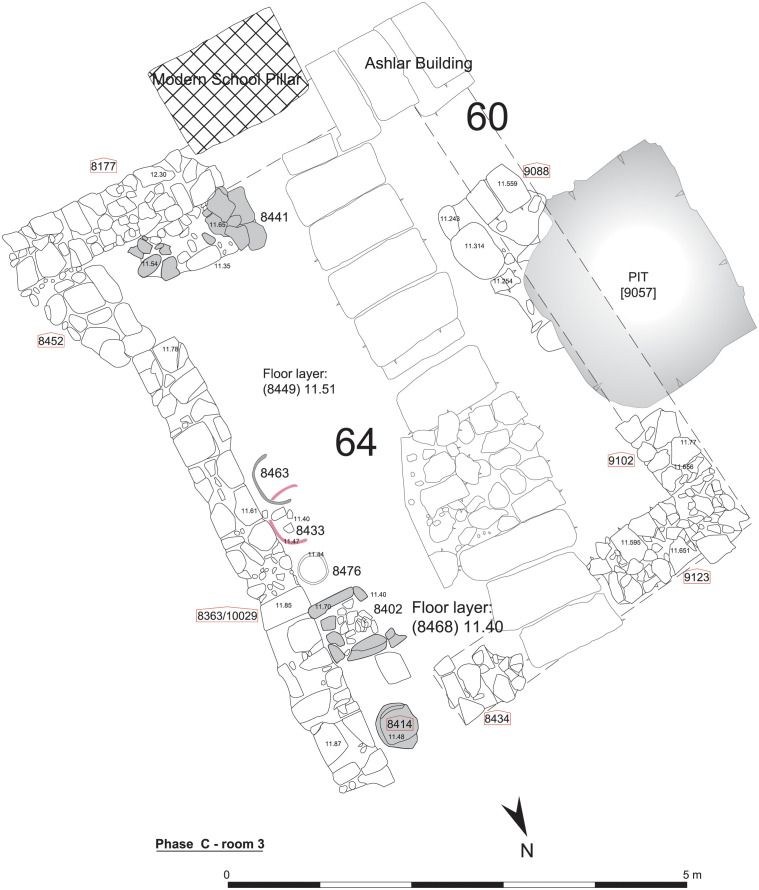
Room 3, Phase C.

**Fig 5:**
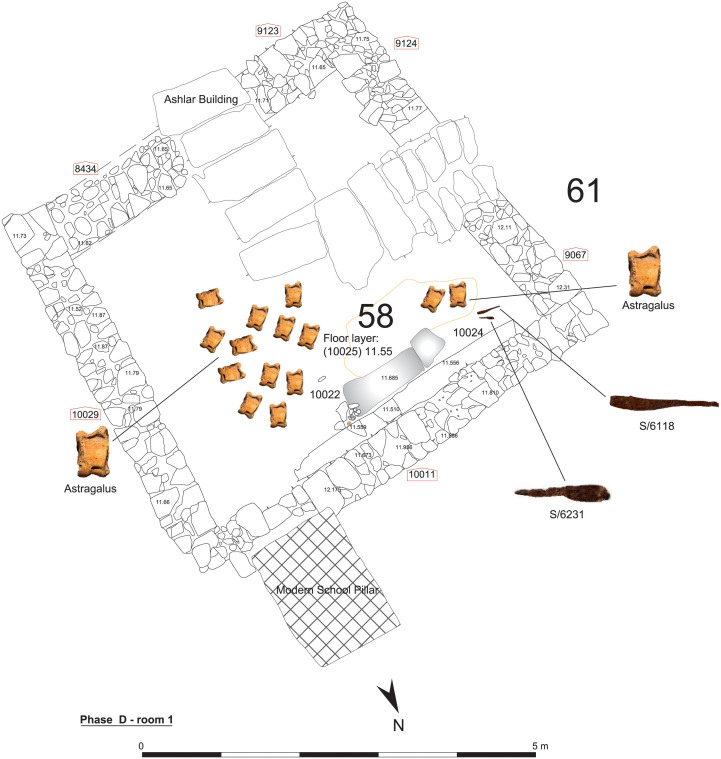
Room 1, Phase D.

Finds in some of the rooms supplied a better understanding of the use-of-space in the building. For example, Room 3 (Fig 4) was equipped in Phase C with tannours constructed against a wall suggesting that the room was associated with the preparation and offering of food. Lastly, there was a pillar sunk into its floor, with only the top visible. The next Phase D, Room 1 was equipped with a bench and a grinding apparatus, while two Iron Age knives were found lying on its floor together with numerous astragali (Fig 5). Micromorphological analysis in the Room 1 during Phases C to E showed that this area, initially a ‘semi-external space’, was occasionally plastered to form an extension of the building’s ‘internal space’.

**Fig 6:**
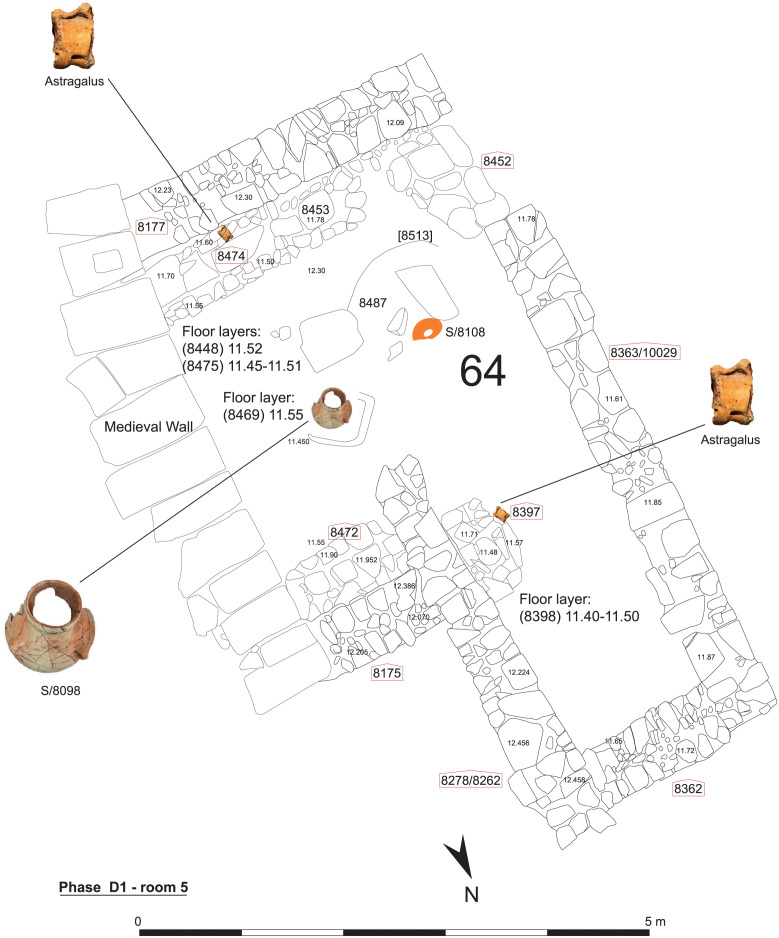
Subphase D1, Room 5.

Room 5 was particularly important for the function of the building in subphase D1 and was equipped with a niche, two benches-along-the-wall and an altar (Fig 6), while Room 6 seems to have been used as a partly open courtyard. Lastly, Room 9 accommodated large pithoi suggesting that this and Room 10 were used specifically as storage facilities. Further details of the stratigraphic sequence from Phase A to G are provided in the publication of the building at Sidon’s College site [12].

### Sampling and ^14^C-Dates

Our samples for AMS-dating were obtained on short-lived organic material from secure and stratigraphically well-defined contexts. As secure contexts, we appraise archaeological assemblages that did not contain later archaeological material, i.e. ceramics that obviously intruded from later into earlier phases. Archaeologists involved in the excavation of multilayer settlements sites are aware of the appearance of residual material through reworking due to levelling and building activities, including the opening of pits. It was due to such activities that sparse pottery finds of Mycenaean type or origin appeared throughout the Early Iron Age stratigraphic sequence of Sidon. This applies in particular to our ^14^C-dated Phases C, D and E. It is not a coincidence that more than half of the Mycenaean and other Late Bronze Age pottery sherds from the site were found in just one room (Room 6), and this is the room that experienced the most extensive reworking. We also have instances of vessels that were reconstructed from sherds scattered throughout different phases. The large majority of the Early Iron Age contexts containing substantial residual ceramic material were not considered to have any value for the purpose of ^14^C-sampling.

To establish an absolute time-scale for Sidon Phases A-K we have at our disposal altogether 37 ^14^C-ages on (annual-growth) olive stones and (short-lived) animal bones (Table 1). A first set of ^14^C-ages were processed in the year 2017 on 29 olive stones and 3 bones at the Oxford ^14^C-AMS laboratory (Lab Code: OxA). A second set of ^14^C-ages on 5 bones were processed in 2018 at the Mannheim ^14^C-AMS laboratory (Lab Code: MAMS). The majority of ^14^C-ages have standard deviations of ± 30 BP or better. According to the information provided by the laboratories, the bone collagen was extracted using weak acid dissolution, followed by ultrafiltration and separation of the fraction > 30 kD. The extracted organic carbon was then dry-frozen and burnt in an Elemental Analyser to produce CO_2_ which was catalysed to produce graphite. The charred olives were pre-treated using the standard ABA-method (Acid/Base/Acid; HCl/NaOH/HCl). By selectively dating olive stones (78%), whenever available, and with sampling extended to include short-lived animal bone (22%) when olive stones were not available, the idea is to avoid any kind of inbuilt ‘old wood’ effect that might be due to any perchance selection of inner tree-rings, reworked wood, or of recycled charcoal e.g. for domestic heating purposes. In consequence, potential outliers are most likely caused by stratigraphic reworking (olive stones), in addition to potential chemical alteration (bones).

**Table 1:** Sidon radiocarbon ages (N = 37).

ID	LabCode	^14^C-Age	Material	Phase	Depth	Room	Context	Calendric Age
		[BP ± 1σ			[m]			[calBC ± 68%]
1	OxA-32124	2909 ± 28	Olive stone	Phase C	11,38	Room 1	Context 10032	1100 ± 50
2	OxA-32125	2870 ± 28	Olive stone	Phase C	11,5	Room 1	Context 10033	1040 ± 50
3	OxA-32122	3443 ± 28	Olive stone	Phase C1	11,45	Room 1	Context 10031	1760 ± 70
4	OxA-39207	2912 ± 21	Olive stone	Phase C1	11,45	Room 1	Context 10031	1100 ± 50
5	OxA-39472	2908 ± 19	Olive stone	Phase D	11,55	Room 1	Context 10025	1090 ± 40
6	OxA-39473	2884 ± 19	Olive stone	Phase E	11,6	Room 1/3	Context 10019	1060 ± 40
7	OxA-34854	2855 ± 28	Olive stone	Phase A	11,22	Room 3	Context 8503	1020 ± 50
8	OxA-39250	2929 ± 20	Olive stone	Phase B	11,26	Room 3	Context 8506	1130 ± 50
9	OxA-39252	2914 ± 20	Olive stone	Phase C	11,4	Room 3	Context 8402	1100 ± 50
10	OxA-39205	2909 ± 21	Olive stone	Phase C	11,4	Room 3	Context 8468	1090 ± 40
11	OxA-34853	2909 ± 26	Olive stone	Phase C	11,51	Room 3	Context 8449	1100 ± 50
12	OxA-35838	2880 ± 29	Olive stone	Phase I	12,15	Room 1/3	Context 8273	1060 ± 50
13	OxA-35837	4145 ± 34	Olive stone	Phase J	12,27	Room 1/3	Context 8259	2740 ± 90
14	OxA-39201	2965 ± 21	Olive stone	Phase A	11,96–11,04	Room 5	Context 8557	1180 ± 40
15	OxA-39206	2834 ± 21	Olive stone	Phase D1	11,35	Room 5	Context 8486	980 ± 40
16	OxA-34851	2823 ± 26	Olive stone	Phase D	11,27	Room 5	Context 8484	970 ± 40
17	OxA-34855	2859 ± 27	Olive stone	Phase D1	11,08	Room 5	Context 8513	1020 ± 50
18	OxA-39203	2922 ± 21	Olive stone	Phase D	11,42	Room 5	Context 8451	1120 ± 50
19	OxA-39204	2874 ± 21	Olive stone	Phase D1	11,78	Room 5	Context 8453	1050 ± 40
20	OxA-34850	2781 ± 27	Olive stone	Phase D	11,46	Room 5	Context 8478	930 ± 40
21	OxA-39471	2823 ± 19	Olive stone	Phase D2	11,58	Room 5	Context 8438	970 ± 30
22	OxA-35663	2840 ± 28	Olive stone	Phase D1	11,45	Room 5	Context 8398	990 ± 40
23	OxA-35662	2884 ± 28	Olive stone	Phase E	11,69	Room 5	Context 8422	1060 ± 40
24	OxA-38490	2792 ± 22	Olive stone	Phase I	12,05	Room 5	Context 8305	940 ± 30
25	OxA-39251	2944 ± 20	Bone	Phase C	11,35	Room 6	Context 4496	1160 ± 40
26	OxA-39202	3071 ± 21	Bone	Phase C1	11,38	Room 6	Context 4503	1340 ± 40
27	OxA-39208	2920 ± 21	Bone	Phase D	11,55	Room 6	Context 4472	1120 ± 50
28	OxA-39209	2897 ± 21	Olive stone	Phase D	11,57	Room 7	Context 7071	1070 ± 40
29	OxA-35665	2792 ± 27	Olive stone	Phase E	11,6	Room 9	Context 8399	940 ± 40
30	OxA-39469	3068 ± 19	Olive stone	Phase C1	11,47	Room 10	Context 8456	1340 ± 40
31	OxA-39470	2907 ± 19	Olive stone	Phase D	11,58	Room 10	Context 8405	1090 ± 40
32	OxA-35664	2933 ± 29	Olive stone	Phase E	11,7	Room 10	Context 8407	1130 ± 60
33	MAMS-36955	2969 ± 28	Bone	Phase C	11,42	Room 3	Context 8485	1180 ± 50
34	MAMS-36956	2770 ± 27	Bone	Phase I	12,02	Room 1,3	Context 8304	910 ± 50
35	MAMS-36957	3078 ± 24	Bone	Phase I	12,05	Room 3	Context 8290	1340 ± 40
36	MAMS-36959	2803 ± 24	Bone	Phase I	12,02	Room 1,3	Context 8315	950 ± 30
37	MAMS-36961	3025 ± 25	Bone	Phase K	11,9	Room 2	Context 9211	1290 ± 60

#### The Sidon ^14^C-Data: Table

The data Table 1 provides in its first column (ID), a running number (ID1 to ID37) for each of the 37 ^14^C-dated samples. These IDs are used, below, to abbreviate the identification of dated samples (e.g. ID1 = OxA-32124: 2908 ± 28 BP). Then follows the laboratory identifier (LabCode) and measured 14C-age. The following columns show the dated MATERIAL (olive stone, bone), the PHASE (A, B, C, C1, D, D1, E, I) to which the sample is attributed, the sample DEPTH [m], the ROOM number from which the sample was taken, and the excavation CONTEXT number. The last column contains the calibrated age with units [calBC ± 68%], calculated by CalPal-software based on IntCal20. This notation is used in preference of multiple calendric age intervals, for which unitary probabilities are often wrongly assumed (see below).

#### Analytical methods

**Fig 7:**
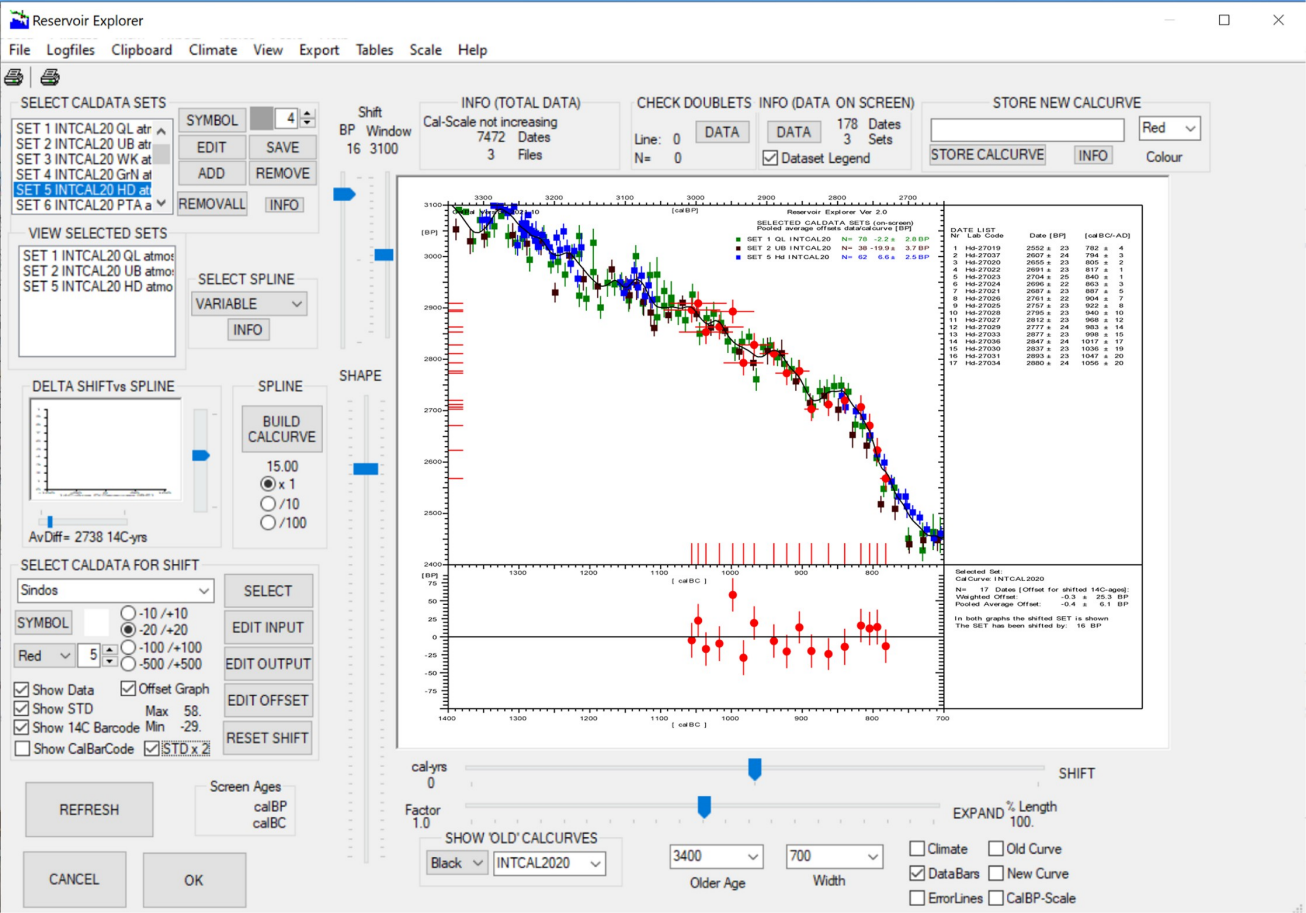
Screenshot of the new CalPal dialog used in the present studies for construction for all site chronologies (Sidon, Megiddo, Tel Rehov, Tel Tayinat). The new graphic technology is illustrated here for the previously published Sindos data set [6]. For purposes of quality control, the construction of site chronologies is performed in parallel to automated ^14^C-offset analysis.

CalPal calibration software is programmed in Win10^®^ and uses Intel^®^ Visual Fortran Intel^®^ VS64 Compiler XE in combination with Winteracter^®^ (Version 14.1) for archaeological ^14^C-age modelling. Once the chronological results have been established by Gaussian Monte Carlo Wiggle Matching (GMCWM), for which we use Fortran IMSL6.0^™^; GAMMQ, CSNDF non-central χ^2^ routines [6]; for the final graphic processing we routinely make use of the CalPal-dialog shown in (Fig 7).

This dialog provides further analytical and graphical functions, which can be used to enhance the already completed chronology, as well as for additional chronological studies. These functions include (1) the construction of a new calibration curve that allows insertion of the recently modelled dataset (for which we use cubic splines with variable stiffness, with polynomial representation of curve variability; in Fortran IMSL6.0^™^; CSSMH, CSVAL; SPLEZ routines), (2) the age-shifting on both scales (^14^C-and calendric) of the different curve construction component files, (3) the graphic addition of these files as well as previously modelled archaeological site ^14^C-chronologies to the study graph, (4) the recently established ^14^C-offset functionality (described below), and (5), the new Miyake functionality. The need for automation of all these different tasks is motivated by the rapidly increasing number of annual tree-ring atmospheric ^14^C-records, the general requirement for more precise archaeological ^14^C-age models, as well as need for more sensitive control on laboratory intercomparisons. Many of these tasks are long-established (e.g. calibration curve construction and interlaboratory calibration differences). A younger topic is the mounting evidence for some small but substantive regional offsets in contemporary (annual) atmospheric ^14^C-levels, as well as seasonal (intra-annual) ^14^C-variability. The study of these offsets, that have strong implications for the increasingly attempted decadel-scale fine-tuning of ^14^C-based site chronologies in our study regions, is now rapidly advancing, thanks to dedicated research by Sturt Manning and colleagues [17, 18].

#### Miyake events

**Fig 8:**
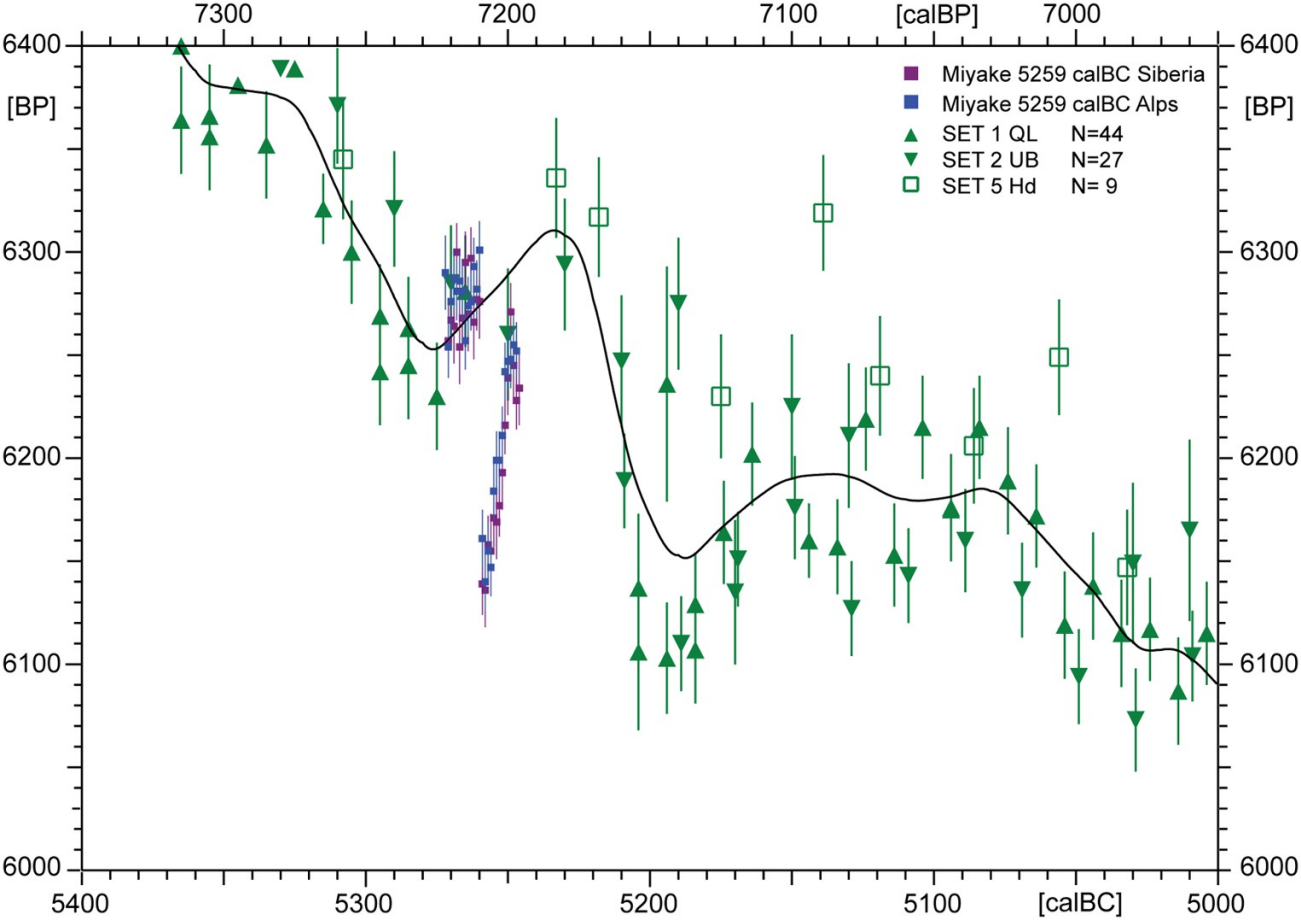
^14^C-data for the 5259 BC Miyake [19] measured on single tree rings of Siberian Larch (lila) and Alpine Larch (blue) compared to IntCal20 sets (green). The graph illustrates how difficult it can be to recognise Miyake events. Despite large amplitude (~ 100 BP), the excess ^14^C is rapidly (within ~20 yrs) removed from the atmosphere. In consequence, the 5259 BC Miyake is hidden from the view of decadel/bidecadel IntCal20 tree-ring data. For clarity the IntCal20 data (from American and European trees) are shown without calendric scale error bars.

A further important and novel topic in its own right is the discovery of solar-proton induced nuclear reactions in the Earth’s atmosphere, whereby large amounts of ^14^C are instantaneously (within a few hrs/days), or over successive years, produced at higher altitudes. As usual, this ^14^C is absorbed in the oceans within a few decades. At present there is evidence for Miyake events at ~ 7176, 5480, 5410, 7156, 3372, 800 and 660 BC [19–23]. We list here a selection of the known (or suspected) events only for the BC-era. The discovery of so many ^14^C-excursions raises the question, how to account for such dynamic atmospheric ^14^C-variability in probabilistic ^14^C-calibration? An equally important question, however, is how to reliably identify the occurrence of a Miyake event, in any given time-window? That this is no easy exercise is illustrated by the efforts invested in the development of software for automated Miyake identification [24]. At present, next to the above-mentioned 660 denBC Miyake event (which occurred during the period known as Pearson’s Peril 800–400 calBC, with largely flat calibration curve), there are no known events for our study period, the Late Bronze and Early Iron Age in the Levant. But we remain suspicious of possible discoveries to come, for reasons that are best explained by way of the following example. In Fig 8 we illustrate the quite extraordinary strategy that is used by the 5259 BC Miyake event in hiding itself from the view of therein unsuspecting archaeologists, in this case for the early Linear Bandkeramik culture (LBK).

**Fig 9:**
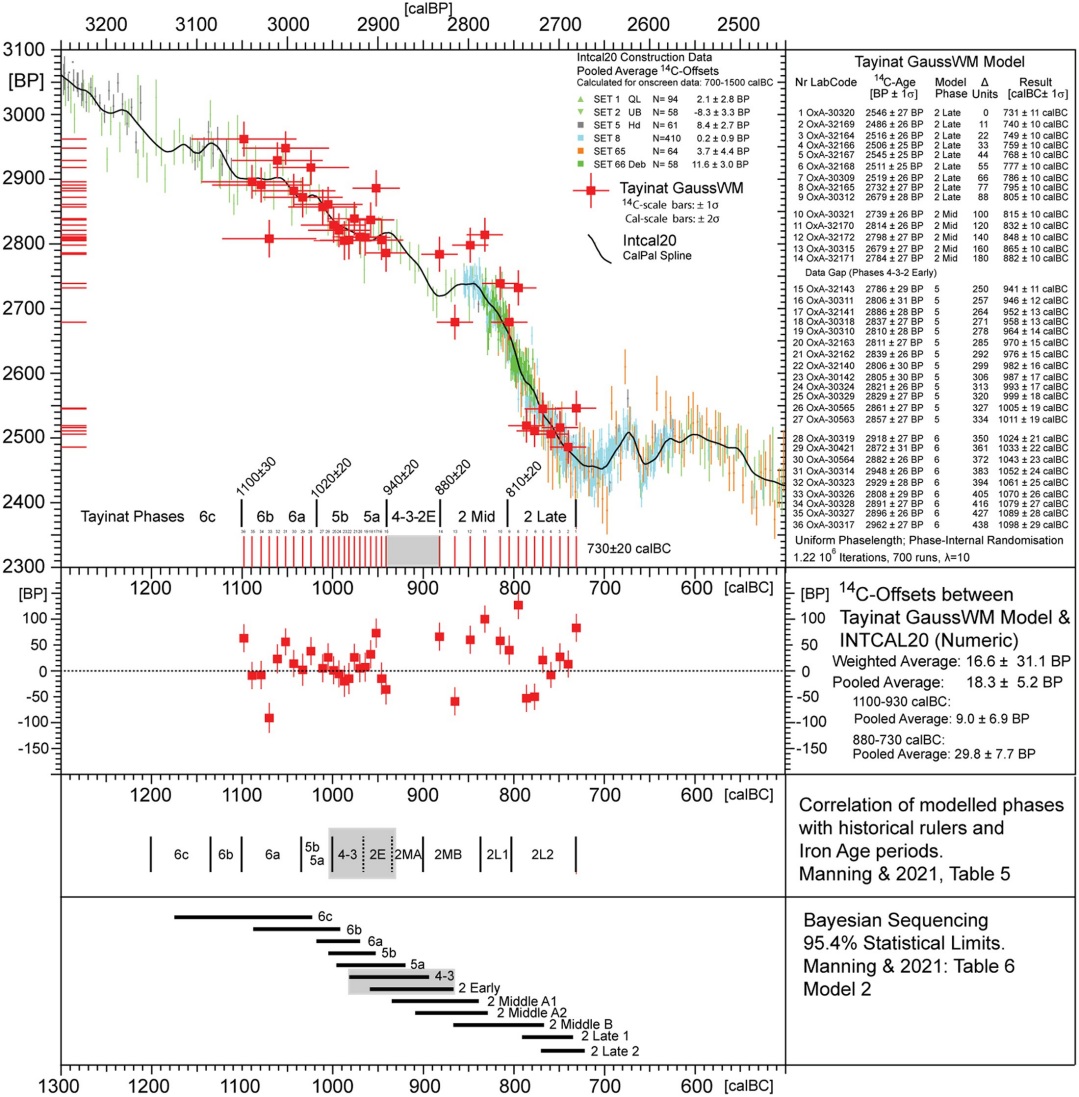
Fig.X3 GaussWM-chronology for Tel Tayinat, based on the same ^14^C-data (N = 50) as published by [28], and using an equivalent archaeological age-model.

According to Brehm et al. 2021 the 5259 BC Miyake was discovered while investigating unexpected difficulties in producing stable Bayesian chronological models for sites centered on the 53^rd^ century BC [19]. We can accept this beautiful science-historical narrative, although the true cause for the observed convergence problems of the Bayesian algorithms [25] is probably not the new Miyake, but–we propose–the use of product-probabilities on the calendric time-scale in the context of multiple re-entry calibration curve wiggles. Namely, given that the existence of the 5259 calBC Miyake event was not only unknown to the archaeologists, but also to the Bayesian algorithms, it can hardly have effected their convergence properties. Our alternative explanation is that, for ^14^C-sequencing at high-resolution we may naturally expect products of probabilities to become progressively inefficient, with increase in dating precision. This is because the number of multiple-readings on the calibration curve wiggles increases faster than the subset of correctly overlapping probabilities, in which case adding further ^14^C-ages is superfluous and further sampling investments may even become contra-productive. Finally, when close to zero-overlap of dates and wiggles, and with calculated *product* probabilities becoming smaller and smaller, the limit of convergence is identical with the collapse of the algorithm. This is described in Weninger et al. 2011, fig. 8 [26]. In comparison, this problem does not occur when using summed probabilities on the ^14^C-scale [27]. The complementary character of such problems, as well as their solution by domaine-switch, are typical for a Fourier Transform (cf. next section). We have tested the validity of this hypothesis for the three study sites (Tel Rehov, Megiddo, Tel Tayinat) that we also use in the pottery studies. At all three sites, the chronological results based on GaussWM (using probability sums in the ^14^C-domaine) are for all practical purposes identical with those based on Bayesian Sequencing (using probability products in the calendric domaine). There are some remaining deviations (beyond noted statistical errors), in the order of a few decades, but–as can be most clearly recognised at Tayinat (Fig 9)–these are most likely caused by translation problems between the different statistical methods and terminologies (e.g. use of point *vs* interval dates; numeric abbreviation of continuous distributions; interpolation for missing data).

In support of ongoing ^14^C-calibration research, we have added new functions to CalPal, including a dialog called ‘Delta ^14^C-Composer’ that is needed to transfer the Miyake events (which are typically published on the Δ^14^C-domain) onto the ^14^C-BP-scale, as well as vice-versa. The CalPal Δ^14^C-dialog utilizes data import/export in Excel^™^-format through the Windows^®^ ODBC^®^-interface, and which supports (user-convenient) spreadsheet-processing. This database functionality makes extensive use of Winteracter^®^ libraries.

#### ^14^C-reservoir and interlaboratory offset analysis

Furthermore, as already illustrated above (Fig 7) and now described in more detail, we have recently added a substantial number of new functions to the CalPal Reservoir Explorer dialog. These functions significantly extend the applicability of the Reservoir Explorer for research towards interlaboratory offsets, interregional reservoir effects, and interannual ^14^C-variability. In brief, the dialog is equipped with two pulldown menus, one of which (Top Left: SELECT CALDATA SETS) allows choice and simultaneous display of a large number (presently N = 168) of data sets (including the available N = 32 Intcal20 sets), whereas the second pulldown menu (Lower Left: SELECT CALDATA FOR SHIFT) offers the same data menu, but with optional selection of only one set (at-a-time). This set is then defined as ‘active’ for requested vertical or horizontal data shifts on the two scales (^14^C- and calendric), based on two corresponding trackbars. The time-series of shifted data is shown in ‘real-time’, such that the user can visually study, for example, the compensating effects of simultaneous ^14^C- and calendric scale shifts. The effects of switching between different calibration curve are described in more detail, below. A particularly important feature of the extended dialog is that the offset-calculations are automatically adapted to the chosen (study-specific) time-window. That is to say, for data sets that extend beyond the screen-window—and which are hidden from the user’s view—the window-boundaries are taken as data-filter. Furthermore, the new dialog supports (left-mouse) cursor-measurement of screen-ages (^14^C- and calendric), with numeric precision of 1 year (depending on window).

The newly developed software allows us to re-address the variability of modern (19^th^ century) atmospheric ^14^C-levels as first studied in Egypt [29] and more recently in the Southern Levant [17]. Both groups address the important and indeed realistic possibility question whether, for example, inter-annual growth season differences that can be measured today (even if only made plausible) may have effected the ^14^C-dates for archaeological samples in the past? The same applies to interregional differences in atmospheric ^14^C-levels, or known variability in reservoir effects. Following Manning et al. [18], although presently known ^14^C-offsets typically have only ‘modest’ ^14^C-amplitudes (in the range of ± 20 ^14^C-yrs), due to calendric-scale ‘amplification’ of these values by the calibration curve, we are well-advised not to underestimate their chronological impact. This applies similarly to the many different kinds of ^14^C-offsets, including the solar-activity induced Miyake events just as much as CO_2_ circulation changes due to past climate-change. Beginning with present studies, and with interest in archaeoclimatology, we have therefore decided to run all archaeological ^14^C-models, as well as associated calibration data (set-by-set), through a fixed schedule of systematic Offset-Analysis. For IntCal20, IntCal13, and IntCal09 the necessary legacy data is available from www.intcal.org. The main components of the schedule are (1) to show the ^14^C-offsets in the time-window under study as graphic time-series, (2) to document the offsets for study data both as weighted averages and as pooled weighted averages, and (3) extend the existing CalPal graphic screen-shading toolbar for Heinrich and Rapid Climate Change events to show the Miyake events. Calculations of ^14^C-offsets are based on Ward and Wilson 1978 and Geyh 2008 [30, 31]. The algorithms were tested for compatibility by comparisons with Dee et al. 2010 [29] (for modern Egyptian samples), and with Manning et al. 2018, 2020 [17, 18] (for modern S-Jordanian tree-ring data). Exact numeric agreement is achieved in both cases. We supply further details of the numeric procedures below.

Next to the new ^14^C-Offset functionality, the updated Reservoir Explorer continues to support the automated spline-based (variable stiffness) construction of ^14^C-age calibration curves. The applied methodology is of interest for the calculation of ^14^C-offsets. In brief, the construction of calibration curves is achieved with the same two pulldown-menus as used in the offset-application (Fig 7 Upper Left), one of which provides a list of available data sets (with ADD/REMOVE/EDIT options), the other (read-only) menu shows the user which data sets are presently selected. Immediately on selection, the new data set is shown on-screen along with the already selected sets. Useful, in this respect, is the possibility to first construct a newly splined calibration curve, and then immediately use it to calculate the ^14^C-offsets for the entire data package. The software design supports rapid switching backwards and forwards between the different calibration curves, including the one that is newly splined. A first result of our studies is that substantial differences—in the order of 2–3 ^14^C-yrs–may exist between ^14^C-offsets that are calculated for splined calibration curves, in comparison to offsets based on tabulated calibration curve data. Interestingly, numeric deviations in this magnitude were already observed by previous authors, already for tabulated data [18, 29]. In designing the new ^14^C-Offset composer we have, in consequence–and followed the advice of these authors—introduced the same formal procedures as used in OxCal’s Delta_R Function. That is, to be precise, we use the same files (IntCal20.14C, IntCal13.14C, IntCal09.13C), and apply essential the same annual interpolation, whenever needed, for the presently available Intcal 5/10/20 tree ring data blocks. Following extensive tests, we are confident that the new automated (menu-selectable, one-click) ^14^C-offset composer provides results that are identical within two decimal positions with those achieved by OxCal.

Having demonstrated (sub-annual) CalPal-OxCal compatibility, we may now take a closer look at the ^14^C-offsets underlying our study-site chronologies. Of particular interest is an update of the Egyptian/S-Levantine ^14^C-offset from its previously established value of ~19 ± 5 BP (as calculated by Dee et al. 2010 [29] for IntCal09, through 19.4 ± 4.8 BP for IntCal13 to the value of 11.3 ± 5.2 BP (as calculated for IntCal20). We emphasise that the decrease in offset, although substantial, can only be taken as interim result. Potential causes for the decrease–beyond normal statistical variability and possible interlaboratory corrections—include increasing CO_2_-emissions during the Industrial Revolution, as well as influx of ^14^C-depleted CO_2_ from the southern Hemisphere. Interestingly, as can be taken from Table 2, similarly strong ^14^C-offsets as in Egypt and the Southern Levant during the 19^th^ century have been measured in Japanese trees. In contrast, for the study period 1500–700 calBC, the data sets show no similarly large changes as in Egypt and the S-Levant between IntCal09, IntCal13, and IntCal20. With the introduction of IntCal20, calculated ^14^C-offsets are generally smaller than for IntCal09 and IntCal13. The majority of values (88%) are in the range of (max) ± 12 BP, and this applies to both study windows (1500–700 calBC, 1600–1920 AD), and to all geographic regions (N-America, Europe, the Near East). The two exceptions are modern Japanese trees (SET65, N = 77) that have an offset of 19.3 ± 2.7 BP, and the Irish trees (N = 44) from 1500–700 calBC that have an offset of– 13.4 ± 3.8 BP. Of course, these findings do not finally outrule the occurrence of short-term geo/bio fluctuations, and which might even be substantial (including Miyake) since they might be smoothened in the time-series by averaging. In conclusion, there is no evidence for the existence of systematic ^14^C-offsets as large as previously deemed possible (i.e. 19 BP). Nonetheless, the data does underline the importance of further studies. We agree, in particular, with the suggestion of Manning et al. 2020 [18] that there may exist brief, but critical periods of variation in Near Eastern regional ^14^C-levels.

**Table 2:** ^14^C-offset results.

Site/Data Set	Sample	Number	IntCal09	IntCal13	IntCal20	Time Interval	Source of Data
	Region	Dates	BP ± 1 σ	BP ± 1 σ	BP ± 1 σ		
Egypt	Egypt	N = 12	19.2 ± 4.8	19.4 ± 4.8	11.3 ± 5.2	1600–1920 AD	Dee et al 2010
Taybet Zaman	S-Jordan	N = 65	18.2 ± 2.5	17.9 ± 1.7	11.7 ± 2.6	1600–1920 AD	Manning & 2020
SET1 QL	Alaska	N = 554	0.2 ± 0.8	0.7 ± 0.8	-5.5 ± 0.9	1600–1920 AD	intcal.org
SET2 UB	Ireland	N = 44	0.0 ± 3.0	5.5 ± 3.4	-4.5 ± 3.2	1600–1920 AD	intcal.org
SET 3 Wk	Ireland	N = 32	14.9 ± 3.6	18.6 ± 3.	10.4 ± 3.8	1600–1920 AD	intcal.org
SET65 Sakamoto	Japan	N = 77	23.6 ± 2.6	23.5 ± 2.6	19.3 ± 2.7	1600–1920 AD	intcal.org
SET69 ETH		N = 388	9.4 ± 0.8	9.3 ± 0.6	3.8 ± 0.9	1600–1920 AD	intcal.org
Tel Rehov	Israel	N = 40	-2.4 ± 5.8	-3.1 ± 5.7	-3.8 ± 6.1	1500–700 calBC	Mazar & 2005
Megiddo	Israel	N = 50	6.3 ± 4.2	0.6 ± 4.0	1.7 ± 4.3	1500–700 calBC	Martin & 2020
Sidon	Lebanon	N = 20	-3.3 ± 6.0	-3.9 ± 5.8	-3.5 ± 6.3	1500–700 calBC	This paper
Tel Tayinat	SE-Turkey	N = 36	-4.7 ± 5.1	-3.5 ± 5.0	-3.6 ± 5.2	1500–700 calBC	Manning & 2020
SET1 QL	Alaska	N = 92	3.0 ± 2.7	-0.2 ± 2.6	0.4 ± 2.8	1500–700 calBC	intcal.org
SET2 UB	Ireland	N = 44	-10.4 ± 3.7	-13.4 ± 3.5	-13.4 ± 3.8	1500–700 calBC	intcal.org
SET 5 Hd	Germany	N = 67	17.1 ± 2.7	8.2 ± 2.4	9.2 ± 2.6	1500–700 calBC	intcal.org
SET 8 UCIAMS	Germany California	N = 278	1.9 ± 1.2	3.9 ± 1.1	1.2 ± 1.1	1500–700 calBC	intcal.org
SET65 Sakamoto	Japan	N = 300	-3.2 ± 8.3	-0.1 ± 8.3	-2.7 ± 8.2	1500–700 calBC	intcal.org
SET66 Debrecen	California	N = 58	16.6 ± 3.0	19.8 ± 2.9	11.2 ± 2.9	1500–700 calBC	intcal.org

#### The mathematics of ^14^C-calibration based on Fourier Transform

In the present study we apply novel concepts of probabilistic ^14^C-analysis based on the description of ^14^C-calibration as Fourier Transform [32]. This needs a brief introduction. The idea is to replace the traditionally classical (as well as Bayesian-classical) concepts of ^14^C-dating probability by a more appropriate description, that we have on loan from quantum physics. As is well-known, concepts of classical physics are not readily applicable to systems of atoms, nuclei, and elementary particles. This applies not only to their mechanical, optical, and other physical properties, but equally to their statistical characteristics. Next to many important changes in experimental and theoretical approaches, the transition from classical physics to quantum physics is pre-eminently accompanied by changes in our understanding of applied statistical concepts, and in particular of concepts relating to key issues such as Uncertainty and Probability, as well their mathematical description. To illustrate these changes, let us take the standard example used in quantum physics. In contrast to classical physics—where the probability of finding a wave at some specific point in time/space is well-described by the wave’s amplitude Ψ—in quantum theory the same (measurable) probability is defined by its squared amplitude Ψ^2^. This allows the wave to have particle properties. The ‘amplitude-squared’ probability concept, described for the first time by Born 1926 [33]. is still today the standard description of so-called ‘wave-particles’. We note that the ‘Born-Rule’ is not only used practically everywhere in quantum physics, but also in electrical engineering, where the energy E transported by a wave with amplitude Ψ is directly proportional to its squared amplitude (i.e. E ~ Ψ^2^). What is presumably unexpected, however, is that the Born-Rule can be adapted to the requirements of ^14^C-age calibration. This is readily possible, for indeed the same reasons as in Quantum physics. The point hereby–historically long hidden from the view of unsuspecting ^14^C-physicists and especially of Bayesian statisticians—is that the mathematical process of ^14^C-calibration is in fact a Fourier Transform. Since the quantum mechanical Born Rule (similarly also the Heisenberg Uncertainty Relation) are also basically a Fourier Transform, it is not at all fortuitous that the same ‘amplitude-squared’ probability concept—once adapted to the purposes of ^14^C-calibration—immediately accounts for practically all known so-called *quantization* properties of archaeological ^14^C-ages (such as clustering, age-shifting, and amplitude-distortion). A presently open question, however, is that—given that ^14^C-calibration is presently only widely accepted as a Bayesian research domaine- in what manner will the clearly existing analogies between quantum theory and ^14^C-age calibration be reflected in the future discussion? As goes for the history of quantum physics, [34] what we observe is a rapid acceptance of the new approaches in the first decade of research (~1925–1935), and this includes their ‘axiomatic’ description [35], followed by a long period of mathematical and epistemological discussion of the new concepts, that is still today continuing. Whatever happens, in archaeology what we surely need in future is the additional clarification of the increasingly complicated concepts used in probabilistic ^14^C-analysis. The very first historically-caused *curiosity* to understand, when we now begin studying these questions from the new perspective of Fourier Transform, is that the dimension [BP] of conventional ^14^C-ages is in fact an inverse calendric time. What this means, in everyday language, is that—if we measure time in seconds [sec]—then ^14^C-ages have physical dimension [1/sec]). It follows that ^14^C-ages [1/sec] multiplied with calendric ages [sec] have the dimension [1/sec]*[sec] = [1]. The same applies when time is measured in calender years [a]: the result of multiplying ^14^C-‘ages’ with calendric ages (on whatever calendric time scale) is always dimensionless. Such ‘beautiful’ properties are at the heart of every kind of Fourier Transform.

The Fourier-based description of ^14^C-calibration begins—as in classical manner—with our well-known representation of any measured ^14^C-age as Gaussian distribution on the ^14^C-scale τ. The aim of Eq.1 is to transfer the measured dating probability from the ^14^C-scale τ to the calendric scale t, for which purpose a calibration curve r(t,τ) is needed.

**Table:** 

$\hat{\text{g}}\left(\tau \right)=\frac{1}{\sigma \sqrt{2\pi }}{\text{e}}^{-\frac{1}{2}{\left(\frac{\mu -\text{r}\left(\text{t},\tau \right)}{\sigma }\right)}^{2}}$	(Eq.1) Gaussian ^14^C-Domain Measurement [BP]

We are using here the following symbols and abbreviations:

τ = Radiocarbon Scale [BP] = [sec^-1^]

t = Calendric Time [sec]

ω = 2πν = angular frequency = [sec^-1^]

^14^C-Age = *μ* ± σ_μ_ [BP]

*r*(*t*, *τ*) ± *σ*(*t*, *τ*) = Calibration Curve [a,BP]

$\sigma =\sqrt{\sigma_{\mu }^{2}+\sigma^{2}\left(\tau \right)}$ [BP]

i = imaginary unit

Although formulated for only one ^14^C-age, that has an assumed Gaussian distribution with width σ and median μ, Eq.1 is easily generalised to allow summation of more than one ^14^C-age. Let us now introduce what in quantum theory is known as a *unitary* operation. As an analogy to preserving the number of wave-particles, the probability assigned to the Gaussians is normalised to an area = 1 on the ^14^C-scale. This unitary operation is achieved according to Eq.2:

**Table:** 

$\int_{-\infty }^{+\infty }\hat{\text{g}}\left(\tau \right)\text{d}\tau =1$	(Eq.2) Normalisation on the ^14^C-scale

Following this measure, which is undertaken prior to ^14^C-calibration, further normalisation of the study function (for example to supposedly account for multiple readings or calibration curve slope variability) is not only unnecessary, it is forbidden. The unitarity operation (*alias* Born rule) as shown in Eq.2 will not be unexpected. However, let us now introduce the Born Rule as fundamental not only to Eq.1, but to all following operations. This is the exact point where the new probabilistic concepts will (presumably) be judged to be misleading, contra-intuitive, strange, or just simply wrong. Translated into everyday language, we are using here the Born Rule as a conservation law for probabilities: once normalised, there is no need to normalise the same ^14^C-data a second time. The next step is to introduce the ^14^C-dates as ‘wave-particles’, for which purpose we need two equations, simultaneously. We are using here a Fourier Transform with two paired equations which are constructed to have unity-product:

**Table:** 

$\text{g}\left(\text{t}\right)=\int_{-\infty }^{+\infty }\hat{\text{g}}\left(\tau \right){\text{e}}^{-\text{i}\omega \tau }\text{d}\tau$	(Eq.3) Forward Direction
	^14^C-Scale (τ) -> Calendric Scale (t)
$\hat{\text{g}}\left(\tau \right)=\int_{-\infty }^{+\infty }\text{g}\left(\text{t}\right){\text{e}}^{\text{i}\omega \text{t}}\text{dt}$	(Eq.4) Inverse Direction
	Calendric Scale (t) -> ^14^C-Scale (τ)

Note that the two equations are twinned in a sense that—although there are apparently two different functions ǧ(τ) and g(t)–there is in fact only one function, but one which can be viewed *either* from the perspective of the ^14^C-scale (τ), *or else* from the perspective of the calendric time scale (t). Ultimately, both functions ǧ(τ) and g(t) represent the same ^14^C-dating probability, but which is viewed from different perspective.

Let us now take a side-look into quantum theory, where the standard procedure for the introduction of probabilities is to describe–whatever we want to measure—as wave-function with Ψ(x,t). Application of the ‘squared-amplitude’ Born Rule then leads to Eq.5, in which the requested probability p(x,t) is expressed as product of the wave-function Ψ(x,t) and its complex conjugate Ψ*(x,t). We do not have to bother here about the meaning of complex numbers. Remembering (as noted above) that all products of ^14^C-‘ages’ [1/a] and calendric ages [a] have dimensionless values, we can easily translate the probability definition of quantum theory (Eq.5) into the language of probabilistic ^14^C-analysis (Eq.6):

**Table:** 

$\text{p}\left(\text{x},\text{t}\right)=\iint \Psi \left(\text{x},\text{t}\right)\Psi^{⋆}\left(\text{x},\text{t}\right)\;\text{dx}\;\text{dt}=1$	(Eq.5) Born Rule in Quantum Theory.
	p = Normalised probability defined for the product of
	the wave-function Ψ(x,t) and its complex conjugate
	Ψ*(x,t) under Fourier Transform.
$\text{p}\left(\text{t},\tau \right)=\iint \text{g}\left(\text{t}\right)\;\hat{\text{g}}\left(\tau \right)\;\text{dt}\;\text{d}\tau =1$	(Eq.6) Born Rule in Radiocarbon Analysis.
	p = Normalised probability defined for the product of
	the Gaussian function $\hat{\text{g}}\left(\tau \right)$ in the ^14^C-domain and its
	twin associated cal-domain function g(t) under
	Fourier Transform.

Finally, Eq.7 describes the use of the Born Rule to analyse any larger set of ^14^C-ages. Note that only one wave function Ψ is used to describe any number N of individual ^14^C-ages, even though each of these may belong to a different sample. The same mathematical narrative, seemingly strange, is used in Quantum physics when–for example–even quite large systems such as the total universe are described by one wave function.

**Table:** 

$\text{p}\left(\text{x},\text{t}\right)=\text{N}\int_{-\infty }^{+\infty }{\left|\Psi \left(\text{x},\text{t}\right)\right|}^{2}\text{dt}=1$	(Eq.7) Born Rule: Probability Definition.
	p = Normalised probability defined for squared
	amplitude of wave-function Ψ(x,t) with scale
	factor N to cover a finite number of ^14^C-dates.

**Fig 10:**
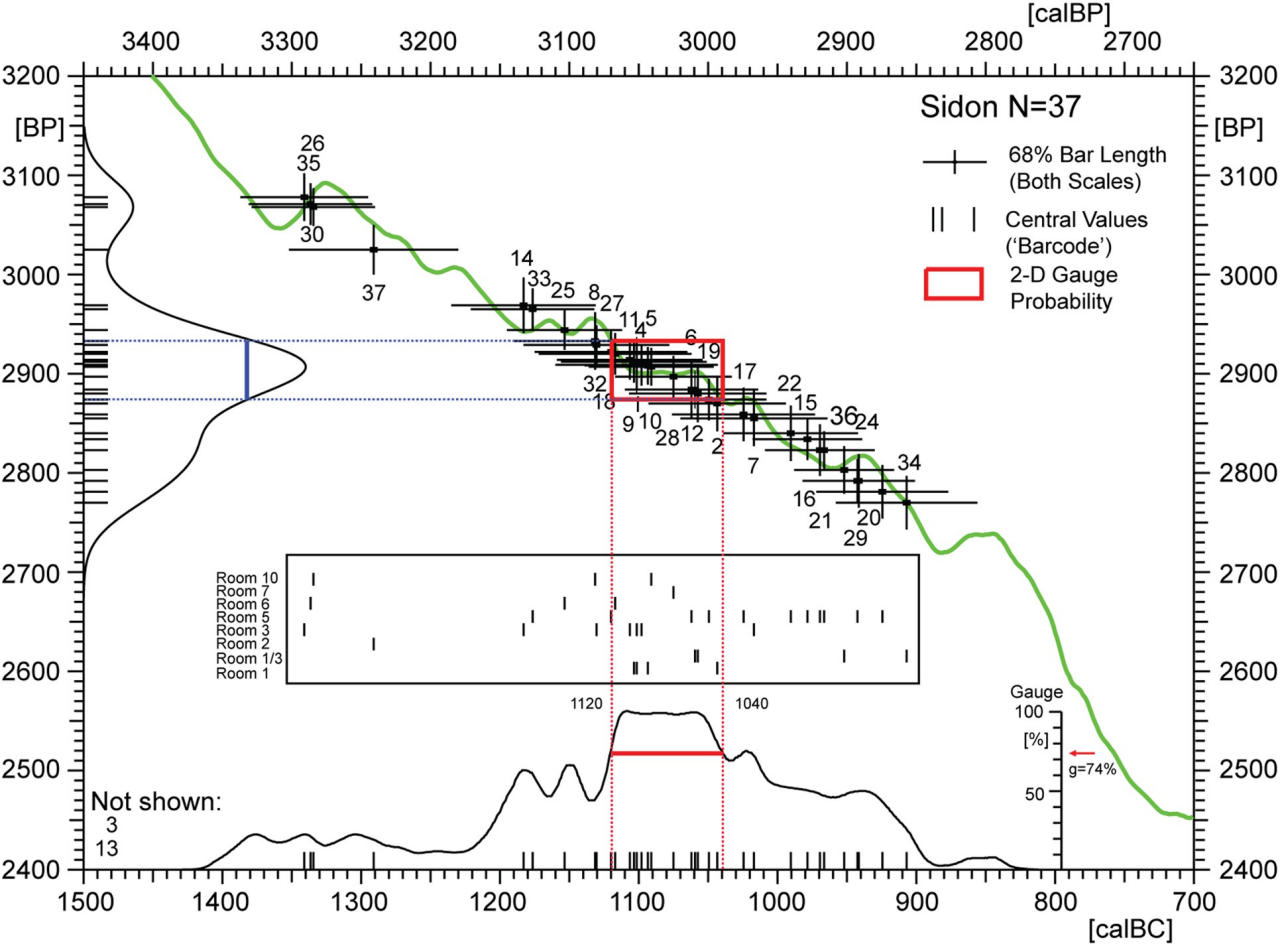
Overview (dispersion diagram) of radiocarbon ages from Sidon (N = 37, Table 1) showing the individual ^14^C-ages as calibrated crossbars with identification numbers (ID1-37). Also shown are the ^14^C-histogram and the summed calibrated 14C-age probability distribution (SPD) of the total data. Calibrated median values of the single 14C ages are shown in barcode representation for total data on the calendric timescale (small vertical lines underlying the SPD envelope). In the centre of the graph the data is re-arranged according to Sidon-rooms (Room 1,1/3, 2, 3, 5, 6, 7, 10). When interpreting such diagrams it important to note that barcode ages (defined as central values of 95%-confidence intervals) are only useful for overview purposes, due to the folding properties of the 14C age calibration curve and related age-distortion (centennial-scale) of calendric ages and probability amplitudes.

As an important corollary of this approach, we recognise that—in order to support the ^14^C-Fourier Transform—the physical dimension of the ^14^C-scale [BP] must (by necessity) represent an inverse calendric time [1/yrs]. This is confimed by an explorative Gedankenexperiment, described in Weninger et al. 2020 [32]. A further consequence of the Fourier approach is a revision in our understanding of where, in the calibration system, the dating probability is positioned. Up until now it has been the general approach to locate the dating probability as ‘area under the summed calibrated distribution’. Instead of changing such long-established concepts (although future studies may show this decision also to be appropriate for Bayesian research), we have defined what is called a ‘gauge probability’. That is easily visualised as ‘plateau box’, namely a rectangular, dimensionless area [[yrs] x [1/yrs] = [1]) that is situated on the calibration curve. What we are undertaking, hereby, is to substitute Born’s amplitude-squared definition of probability (P = ΨΨ*) by multiplication of the ^14^C-scale histogram-width with the height of the associated summed calibrated distribution. Although easy to implement in ^14^C-age calibration software, from a theoretical perspective this procedure (illustrated for Sidon in (Fig 10) amounts to a fundamental re-interpretation of what we may now visualise as ‘true dating probability’.

#### The Sidon-data: Overview

Application of the new concepts provides us with a first overview of the ^14^C-ages from Sidon. As shown in Fig 10, the data can be viewed separately (in a well-known manner) as ^14^C-histogram and summed calibrated distribution on the two scales. Applying the Born Rule, we now use their reinterpretation as wave functions to assign a true dating probability to any requested interval of the calendar time scale. Let us now visualise this probability as a 2D-area (i.e. rectangle, *alias* ‘plateau box’) that is sitting on the calibration curve.

As illustrated in Fig 10 the plateau-box construction is achieved in two steps, first by vertical projection of the calendric-scale study interval (red) onto the calibration curve, second by horizontal projection of the calibration curve crossing points onto the ^14^C-histogram, where–finally—the requested ^14^C-scale interval (blue) is determined. This procedure can be repeated for any study interval. What is specifically shown in Fig 10 is the temporal setting of the dating probability (red square, *alias* ‘2D-gauge probability’) that is simultaneously in common to the central and most prominent parts of the two wave functions. By the same procedure the dating probability can be constructed for any requested calendric scale interval. This includes the entire calendar scale as well as arbitrarily small intervals, although not for dots. From mathematical perspective it is important to note (1), that non-overlapping calendric intervals always have assigned non-overlapping rectangles (and vice-versa), as well as (2), that for gap-free calendric-scale intervals the assigned rectangles are also gap-free (and vice-versa). In graphic ^14^C-analysis these algebraic-geometric properties are useful to know. In a nutshell, based on the well-known Born rule and recognition that ^14^C-calibration accords to a Fourier Transform, we have introduced a number of new theoretical concepts that may be of help in understanding the often ‘curious’ properties of ^14^C-dates encountered in archaeological studies (e.g. the sharp and obviously erroneous Bayesian ‘spikes’ on summed calibrated ^14^C-distributions).

It also follows naturally from its description as Fourier Transform that we should be able to express ^14^C-calibration using Heisenberg matrix multiplication, in which case we could write the ^14^C-type of Uncertainty Relation in Dirac notation:

$$\left[\text{A},\text{B}\right]=\text{AB-BA}\neq 0$$

with A representing a 2D (^14^C & cal-scale) calibration matrix and vector B containing containing the 1D (^14^C-scale) archaeological study data, that need calibration. From the properties of matrix multiplication, it follows naturally that ^14^C-calibration is non-commutative, but that we already know. What we did not know, prior to preparing the present paper, is the actual demonstration (written in R-language) that ^14^C-age calibration can indeed be programmed as matrix multiplication. This is demonstrated in a brief but methodologically crucial archive publication by Martin Hinz [36]. With this important step already taken, we may confidently look forward to the further description of ^14^C-calibration in terms of Bayesian matrix-analysis, and which is apparently also now underway [37].

#### Age distortion vs sampling focus

**Fig 11:**
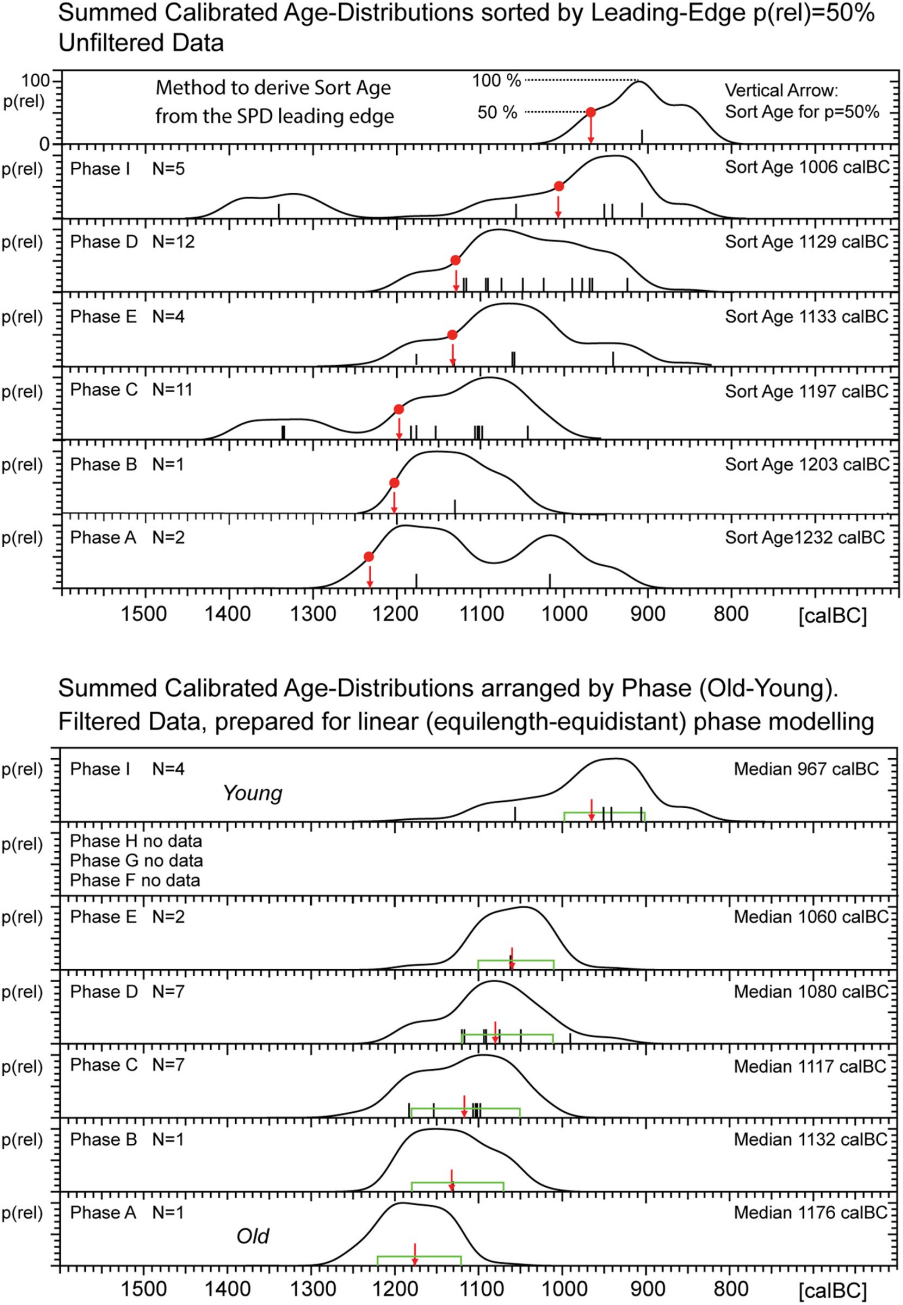
Stratigraphic integrity of Sidon data (Table 1), evaluated by SPD-sequencing. (**Upper Graph)**: The SPDs of each Sidon Phase (A,B,C,D,E,I) are arranged according to their known stratigraphic sequence, from oldest to youngest. The lowest SPD is for Phase A (N = 2 dates), the uppermost SPD is for Phase I (N = 4 dates). The known SPD-order is used to derive a specific SPD-amplitude that is valid for the SPDs of all phases, and which can be used to automatically sort the SPDs. All SPDs are assigned a common max amplitude (p = 100%). Then there also exists a common amplitude of p = 50%, that can be measured on the leading SPD-signal edge, that we can use for automated sorting (not shown). The SPD-specific numeric sort ages are shown on the right. (**Lower Graph)**: In order to achieve a correctly sorted SPD-sequence, alternatively, the same sorting procedure can be applied to the outlier-filtered SPDs. For screening purposes prior to the application of more advanced (but time-consuming) GaussWM modelling procedures, it is (optionally) possible to replace the leading-edge seriation by an algorithm based on trailing-edge or interquartile seriation.

**Fig 12:**
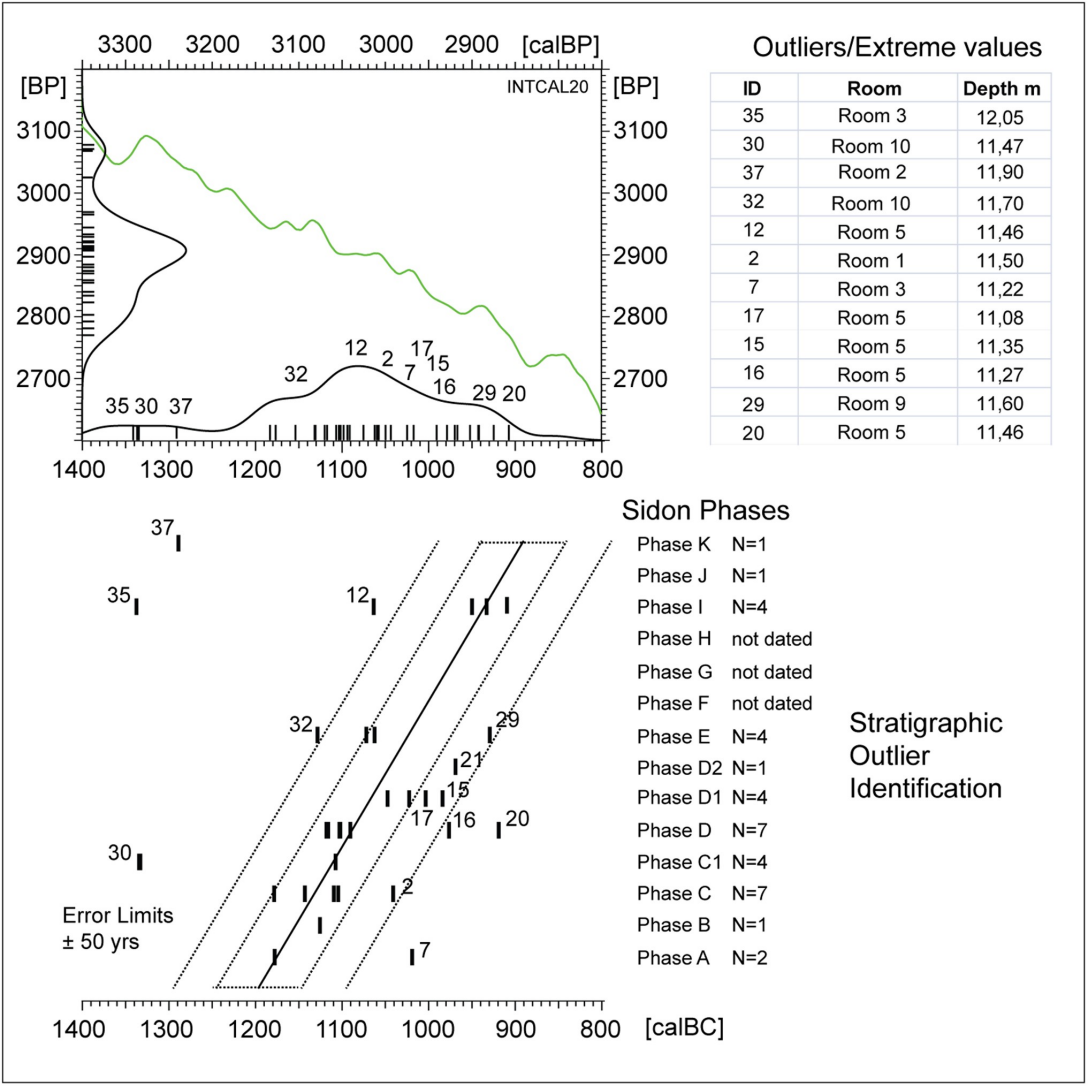
Identification of outliers and extreme ^14^C-ages based on barcode representation of Sidon data (Table 1). In this graph all Sidon Phases (A-K) are included, with allowance for not-dated Phases F, G, and H. (**Upper)**: ^14^C-histogram and calibrated probability distribution of data, numbered according to Table 1, shown in context with IntCal20 (green line). (**Lower)**: the same data, but showing barcodes grouped according to Sidon Phase (A-K), with Phases arranged vertically according to their known stratigraphic sequence. Note, empty vertical spaces are assigned to not-dated Phases F, G, and H. This procedure, in combination with the visual setting of oblique error-lines (dashed lines drawn at ± 50yrs (~1σ) and ± 100yrs (~2σ)), facilitates first-order identification of stratigraphic outliers (sample ID: Table 1). Based on this monitoring, ^14^C-ages on samples with ID 7, 30, 35, 37 should be treated as outliers. Further outliers (better: extreme ^14^C-ages with expected modelling deviations larger than 100 calyrs) are for samples with ID 16, 20, 32, and 12. This remains to be confirmed by explicit modelling. Finally, although to smaller extent, the ^14^C-ages on samples with ID 2, 15, 17, 21, and 29 are also forecasted to show modelling deviations, in this case on the level of ~ 50 yrs. Of special interest is the recognition that, for the youngest Phases I, J, and K, apparently only three of the six dates (i.e. 50%) are likely to be in correct stratigraphic order, and all three of these dates are from Phase I. On the older side of the stratigraphy, with ID7 from Phase A clearly too young, there is only one remaining date for each of two earliest Phases A and B. This we judge is an insufficient number for application of advanced statistical studies.

We turn now to the important questions of outlier-analysis, sampling focus, and quantum age-distortion due to the shape of the calibration curve, which are often taken as three separate topics, but which are better studied together. At Sidon, with the main aim of outlier analysis for the application of higher-resolution age-models, we have applied the SPD-method (Fig 11) in combination with the method of SPD-Barcode-Sequencing (Fig 12). Methods and results are described in the captions of Figs 11 and 12. Although from three different phases (phases C1, I, K), the three oldest ^14^C-ages (ID 30, 35, 37), show a cluster of dates in the 14^th^ century calBC. This is clearly due to stratigraphic reworking of samples from an older event (Fig 12). Turning to the question of age-distortion due to the shape of the calibration curve, the occurrence of a long plateau 1130–1050 calBC is most easily recognisable in the strong barcode cluster at ~1100 calBC. We note that this effect is enhanced (and becomes observable) due to the strong focus on taking samples from different phases of Rooms 1 and 3. The distortion mechanism here is that the sampling focus, in combination with the plateau at ~ 1100 calBC, leads to the strong ^14^C-histogram peak at ~2910 BP, which is then transferred over to the calendric time-scale. Finally, we note the existence of two further plateaus, namely at ~1180–1140 calBC and ~1100–1050 calBC, which are similarly strong, but less extensively sampled.

**Fig 13:**
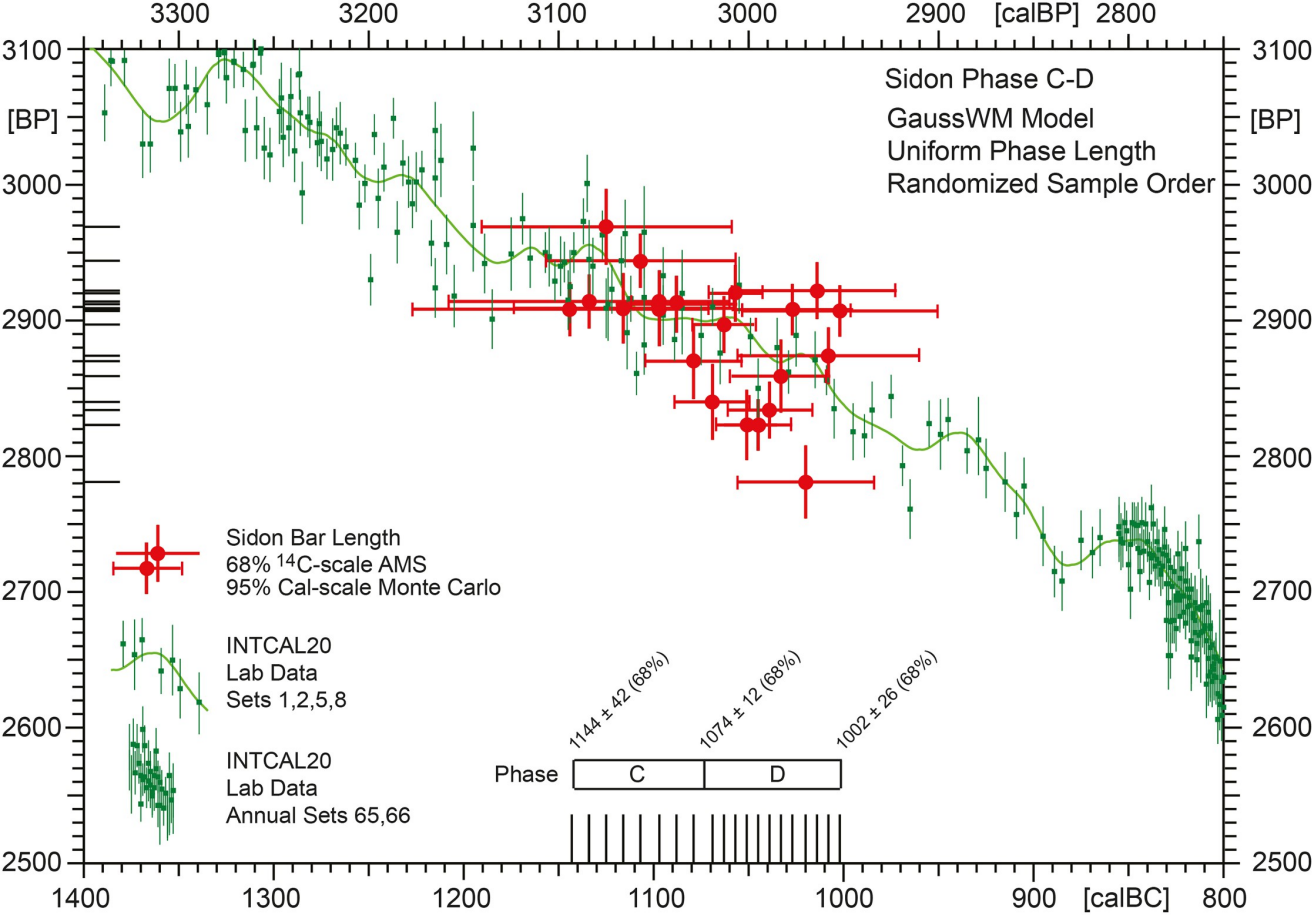
Archaeological uniform phase-length model for Sidon phases C-D, based on N = 20 stratigraphically filtered ^14^C-dates (i.e.with outliers removed from Table 1 according to the outlier analysis, Fig 12. The ^14^C-ages are arranged in the order of the stratigraphic layers from which the samples were taken, with layer-internal randomisation. The applied GaussWM-modelling is based on 1000 runs (run-time 24 hrs) with assumed Gaussian Monte Carlo modelling errors of ± 10 yrs for sample positions, ± 10 BP for calibration curve rebuilding, and application of a non-central chi-squared metric with non-centrality parameter λ = 10. Note that the specific sample-sequence shown as age-model in Fig 12 represents only one of several million alternative sequences, all of which contribute equally to the finally calculated model errors.

**Fig 14:**
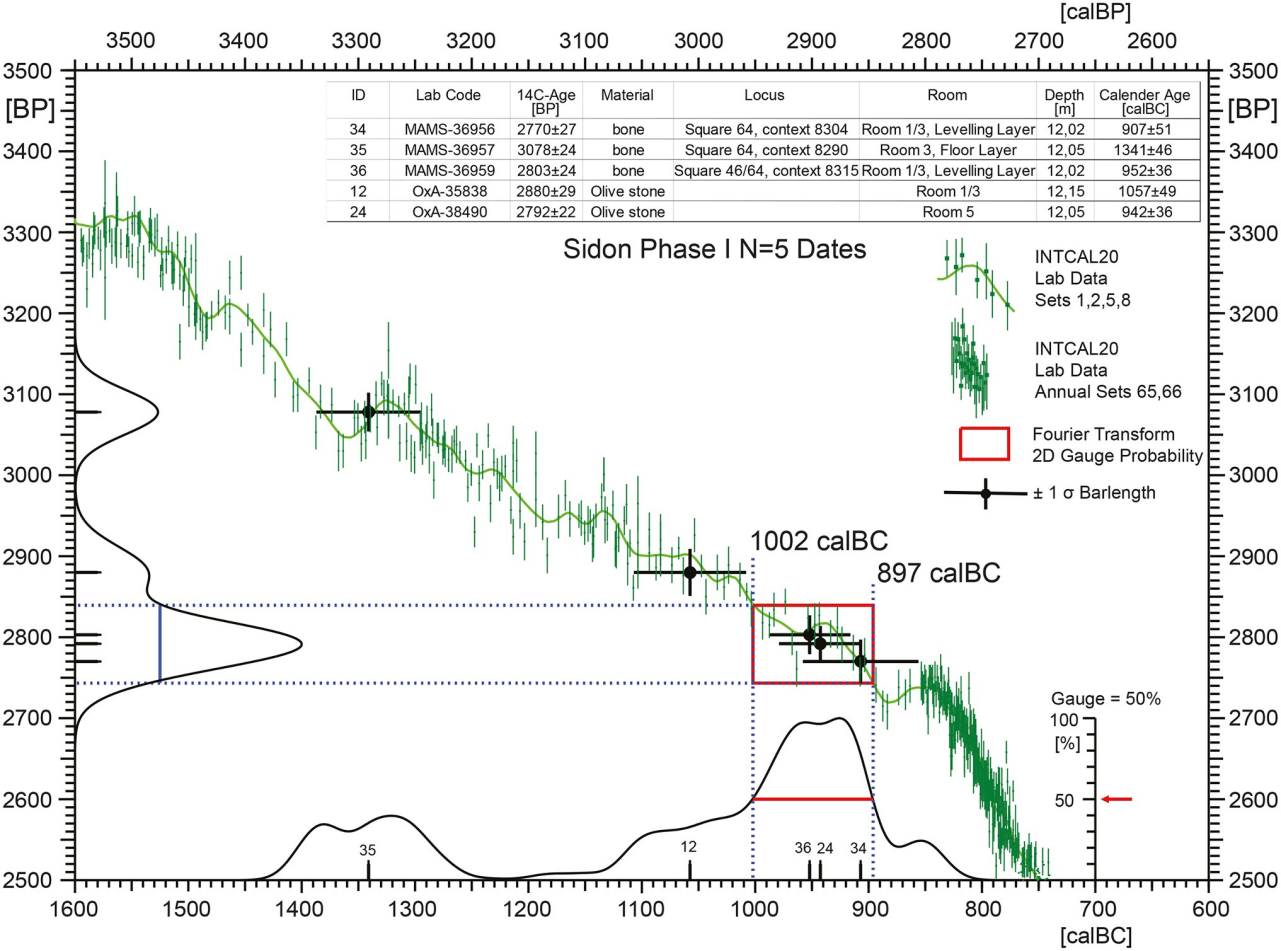
Application of the probability gauge method to Sidon Phase I dates, used to estimate the Begin and End of the phase. The available N = 5 dates are shown as single-age crossbars (± 68% confidence), with sample ID according to Table 1. The red-coloured rectangle projected onto the IntCal20 curve shows the visually optimised central area of 2D-dating probability. The underlying gauge value of g = 50% was chosen in order to enclose simultaneously as much central area of the uncalibrated (^14^C-scale) and calibrated (calendric scale) distributions as possible. The result is that Sidon Phase I begins at 1000 ± 40 calBC and ends at 900 ± 40 calBC (rounded ages, with dating errors estimated from underlying single dates (cf. Table 1).

To conclude, and given that Phases F, G, and H are not dated, in the analysis described below we have constructed and tested an explicit GaussWM age-model only for the remaining N = 20 dates from Phases C and D. This is shown in Fig 13. In a further analysis, described in Fig 14, we use the Fourier method to derive calender ages for Begin and End of Phase I. This study is based on all available N = 5 dates i.e. in this study we do not make of the forecasting that ID 12 and 35 are likely to be outliers, although this expectation is immediately confirmed by dispersion calibration of the Phase I dates (Fig 14).

During runtime the sequence of 14C-ages is incrementally expanded (1 yr steps) parallel to the calendric time scale, at each step with random shuffling of the phase-internal sample position. Such random shuffling has its main use in quantifying the dating errors achieved for individual sample positions (i.e. marginal probabilities). This phase-internal sample-order randomization allows, in the present application, derivation of empirical errors for the begin of Phase C (1144 ± 42 calBC), the transition from Phase C to D (1074 ± 12 calBC), and for the end of phase D (1002 ± 26 calBC).

## Discussion of the pottery evidence

### Local Phoenician ceramic types and sequence at Sidon

For the purposes of maintaining a consistent ceramic typology, only well-defined contexts were selected following a stratigraphic assessment of their contextual properties. Finds from floors in association with architectural features were judged to be acceptable for further analysis as well as artefact assemblages from pits that could be associated with the period of occupation of certain floors. A total of 1267 diagnostic fragments were typologically analysed and assigned to several vessel types such as Platter bowls (PB), Bowls (BL), Jars (J) and Jar or Jugs (JJ), Pithoi (P), Hole Mouth jars (HM), Kraters (KR), Cooking pots (CP), Bases (B), Handles (H) and Lamps (L).

**Fig 15:**
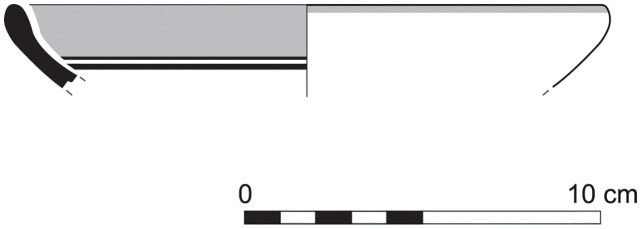
Phoenician Bichrome Ware: S/116259/8308.

**Fig 16:**
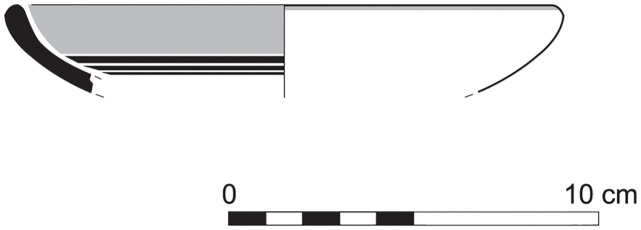
Phoenician Bichrome Ware: S/109470/8324.

**Fig 17:**
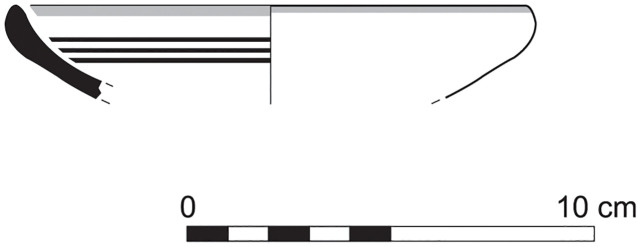
Phoenician Bichrome Ware: S/109455/8324.

**Fig 18:**
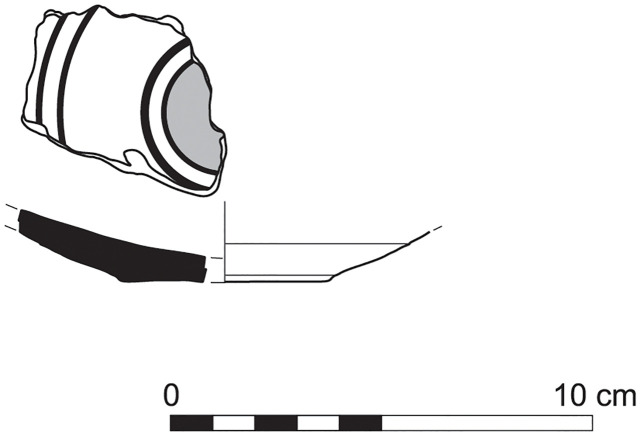
Phoenician Bichrome Ware: S/110604/8322.

**Fig 19:**
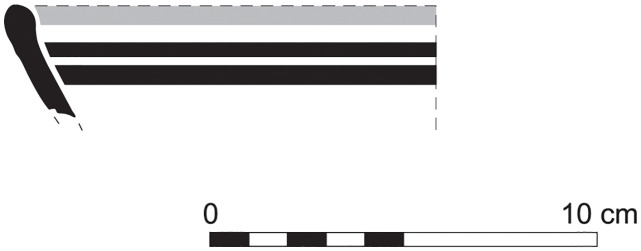
Phoenician Bichrome Ware: S/110605/8322.

**Fig 20:**
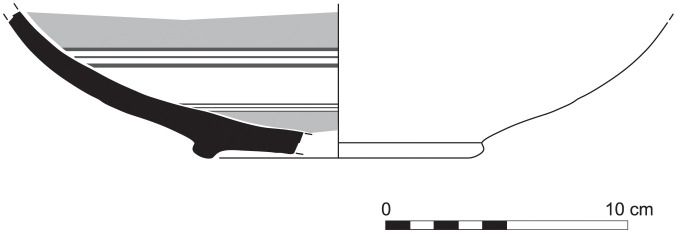
Phoenician Bichrome Ware: S/91639/1008.

**Fig 21:**
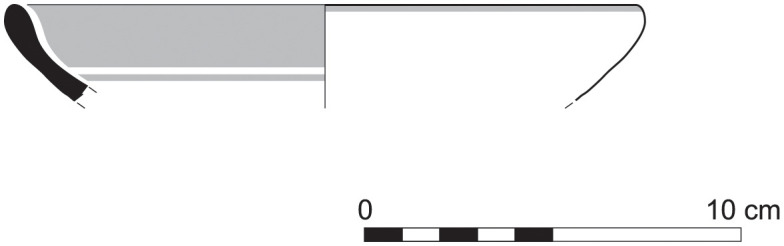
Phoenician Monochrome Ware S/S/131497/8348.

The group of Platter bowls and Bowls constitutes 58.96% of the whole local pottery assemblage. Platter bowls (PB) with their characteristic shallow form were probably used for serving or dining, while the Bowls (BL) are deeper with round or vertical sides. At Sidon, platter bowls were the items most deemed worthy of the investment of bichrome painted decoration (Figs 15–20) [12] with some of these vessels still exhibiting the distinctive monochrome version of the bichrome decorative syntax of wide and narrow bands, (Fig 21) while in Dor, flasks, jars and after that jugs and strainer jugs are the most common containers that were first painted [38].

The JJ group of ceramic forms constitutes 23.28% of the assemblage. As no complete shape was found (except for vessels deposited in a pit, see below), it proved difficult to differentiate between the two types of vessels using only rims classifying them into two groups. The first includes only jars (J) of types 1 to 4, all of which belong to a well-known Levantine family of storage jars with their horizontal sometimes slightly convex sharp carination on the shoulder. The second group comprises rims that may belong either to jars or jugs (JJ). Jars from type 1–4 continue the Late Bronze Age Canaanite tradition with the angular profile of the shoulder endowing the vessels with robustness as well as more resistance to mechanical shock thus allowing, at the same time, for several amphoras to be packed next to each other as well as stacked on top of each other. Protruding knob bases on these jars, also a Late Bronze Age heritage, made these vessels easier to handle as they could be moved and placed on the ground by easily rotating them on their base. These are the most common jar types at Phoenician sites such as Sarepta, Tyre, Tel Keisan and Dor [12]. Finally, painted red-monochrome decoration is more common than black-monochrome in the local pottery assemblage of Sidon. Three fragments [12] may be regional variations of the so-called overlapping multiple diagonal strokes (OMDS). While red slip ware appears more rarely than black-monochrome painted ware, the technique of applying a red coating is found in all phases, becoming more frequent from Phase C onwards and gaining in popularity in Phase G.

**Fig 22:**
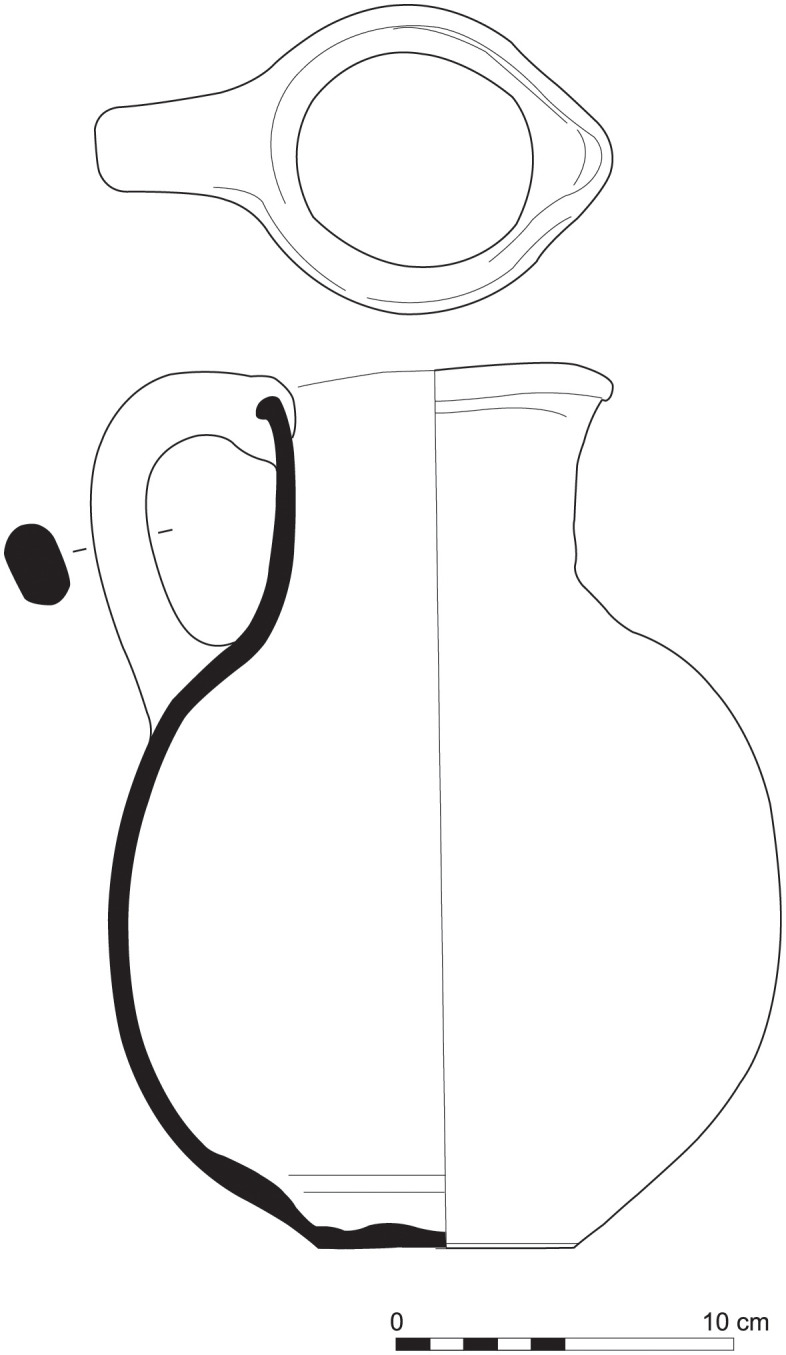
Trefoil mouth jug S/8268/4429.

**Fig 23:**
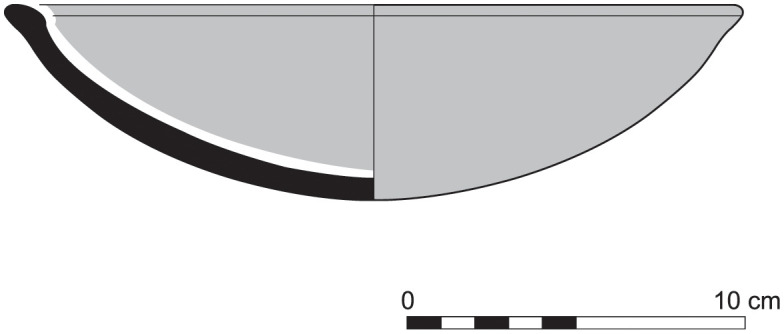
Platter bowl S/8269/4429+4430 adorned with red slip.

**Fig 24:**
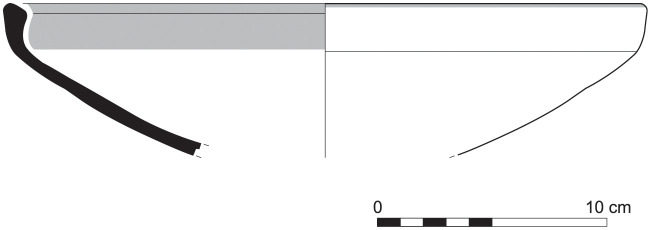
Platter bowl S/8267/4429 painted with a horizontal red painted band on the inner surface under the rim.

**Fig 25:**
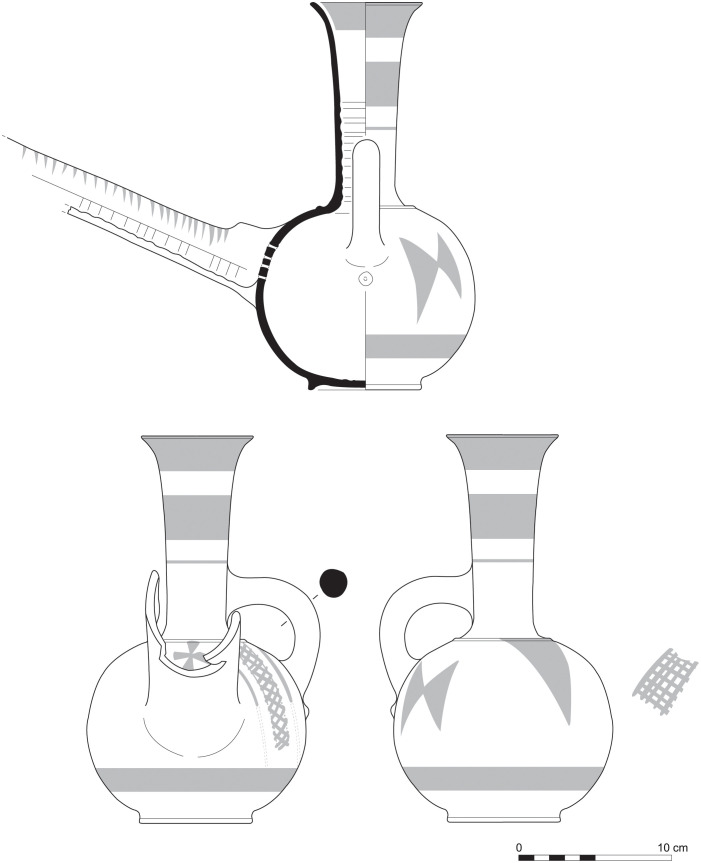
Strainer spouted jug S/8291/4429.

**Fig 26:**
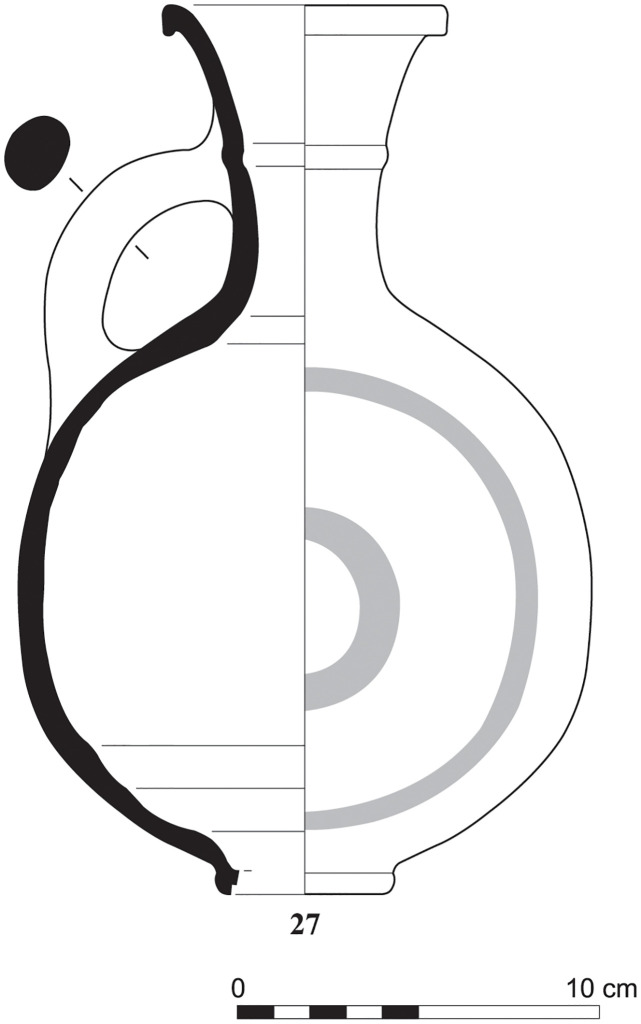
Jug with a ridge on the neck S/8152/4429.

**Fig 27:**
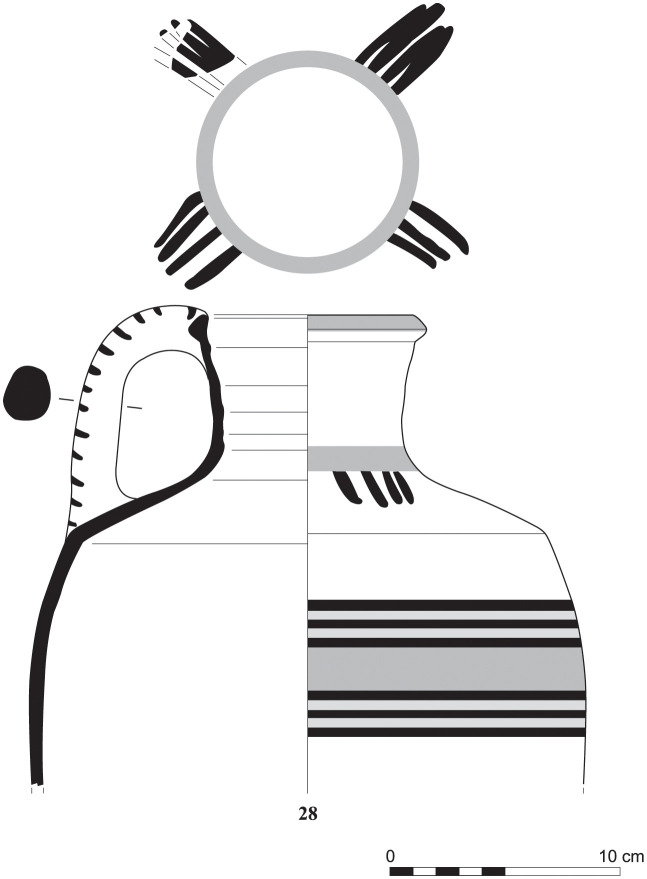
Jug with handle from rim to shoulder S/8154/4429.

With the exception of a trefoil-mouth jug (Fig 22), all other vessels that were deposited in a pit–probably for ritual purposes–belonging to Phase G, were variably decorated. This is the only context from Sidon where platter bowls (Figs 23 and 24), jugs and strainer jugs were painted with monochrome and bichrome Phoenician decoration or adorned with red slip. A strainer-spouted jug (Fig 25) was decorated in the typical manner of the earliest phase of the Phoenician Iron Age ‘Kouklia horizon’. The red painted decorative pattern consists of the ‘butterfly’ motif, a pendent triangle and a vertical lattice band between two lines on the shoulder, while a Maltese cross is painted above the spout [12]. A jug with a ridge on the neck (Fig 26) bears a red painted concentric circle in the earliest red-monochrome tradition instead of the more common bichrome circle without decoration on the neck [12]. Finally, a jug with handle from rim to shoulder (Fig 27) is the only vessel in this context with bichrome decoration consisting of narrow black lines that enclose a red band on the upper body. It also has black painted tassels hanging from a red painted band encircling the neck, while its rim is covered with red paint and horizontal black painted brush strokes cover the handle [12].

The kraters constitute 7.18% of the assemblage. The fragmentary preservation of the pottery did not allow any detailed typology of this shape neither a consistent grouping. The pithoi make 1.26% of the whole ceramic assemblage. A large pithos is the only complete vessel found sunk into the floor of Room 9. Given its height, namely 1.21 m, the vessel was most probably stationary and therefore handleless. Early Iron Age pithoi are fairly uniform in shape and proportion. The Sidon vessel, with a triangular-shaped rim, a vertical neck and a narrow stump base does not bear the typical ‘wavy line’ decoration. Instead, it has three applied simple bands. Similar pithoi without wavy band decoration were also found at Megiddo, Tel Keisan and in Tyre, stratum XV. No less than 9% of the whole local ceramic assemblage comprises cooking pots of different types that display continuity from the Late Bronze Age, almost all of them without preserved handles [12]. Phases A to G yielded 17 chalices as well as 2 goblets and 4 cup-and-saucers. Finally, lamps are abundant in the Early Iron Age ceramic contexts but very fragmentary [12].

In her study of the Iron Age pottery from Dor, Ayelet Gilboa [39] underlines the regional variability of the Early Iron Age ceramic assemblages in Phoenicia. Some pottery shapes from Sidon show strong links to the Late Bronze Age local Canaanite tradition. The local ceramic repertoire of the chronological horizons from Phases A to G embodies continuity throughout. There are still however some changes in the development of some shapes such as platter bowls, whose type PB4/4a/4b declined after Subphase D2, while others such as PB3a, PB5b,c.d, PB6b, PB7, PB10, BL11, BL17a were not particularly common in the earlier phases [12]. The dominance of vessels associated with eating and drinking practices suggests the function of the building as a place of communal feasting and may further indicate the type of ritual consumption practiced there. Phase E marks the beginning of a new cultural as well as chronological phase with a substantial change in the architecture of the building together with the slow emergence, for the first time, of a small quantity of Phoenician Bichrome Ware [12]. The new ceramic style flourishes however in Phases F and G. The first vessels to bear bichrome decoration are open vessels, namely platter bowls and bowls. This new phase does not reflect any sort of break but rather a smooth transition from the previous ones. The Sidon Phase E horizon is key to ceramic synchronisation with other sites. It can be correlated with stratum E in Sarepta, Tyre XIII to the very beginning of Phase XI [8], Tel Dor Ir1/2 [10] and corresponds to Megiddo VIA (area H, str. H-9 and area K, stratum K4) [40].

### The Greek Geometric pottery context

The recent excavations at Sidon have brought to light one of the largest assemblages of Greek Geometric pottery that was ever found along the Levantine coast. On the one hand, this is a significant find that provides new evidence about the social and cultural perception of Greek pottery in the eastern Mediterranean since it comes from a context that was interpreted as ritual. On the other hand, these pottery finds are particularly helpful in the ongoing discussion about the inter-regional correlation of the Aegean and eastern Mediterranean pottery styles as well as to achieve a more widely acceptable consensus in Mediterranean chronology.

Contra to the usual practice in the eastern Mediterranean that every single Greek sherd found at a Levantine site is celebrated as a chronological marker–no matter how insecure its context was, how well it was preserved and how far it could be relatively dated (see below)–at Sidon we have undertaken for the purposes of the present study a strict selection of best-stratified sherds [14, 41]. We use this qualified data below in our discussion on interregional chronological correlations between the Levant, the Aegean, and Greece.

**Fig 28:**
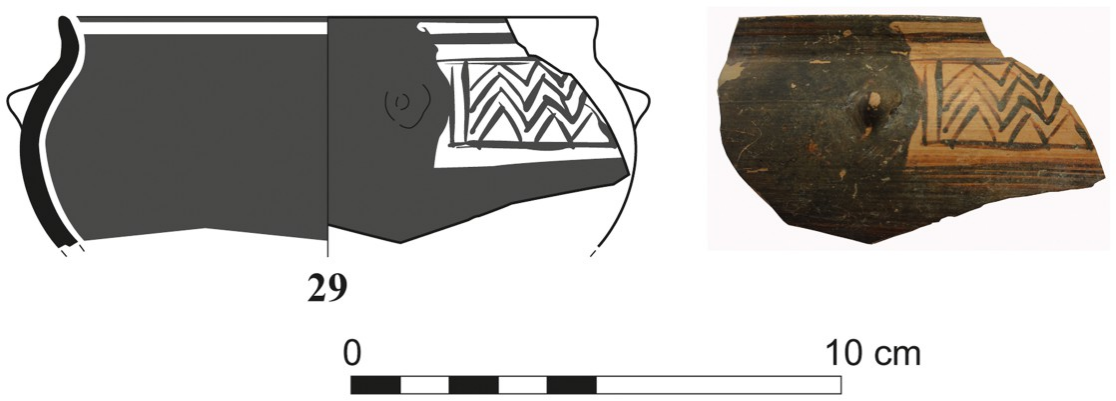
Fragment of a Greek zigzag bowl from Sidon phase D dating to Early Geometric / Subprotogeometric II.

Out of a steadily growing assemblage of currently ca. 55 Greek Geometric pottery sherds from Sidon that date from the Late Protogeometric period onwards, a sherd from a bowl (S/8569/8579)–probably a one-handled cup rather than a skyphos–with multiple zigzag and a plastic wart represents one of the earliest and best stratified fragments of Greek wares at Sidon that was dated to Early Geometric (Fig 28). This sherd was found on the floor of a conspicuous installation comprising two shallow joint plastered circular pits that were used during Phase D in room 7 of the building excavated at the College Site [12]. The fabric of the sherd was macroscopically defined as Euboean that makes this vessel one of the earliest Atticizing bowls of Euboean origin decorated with multiple zigzag lines, as some good parallels from closed burial contexts at Lefkandi suggest (Tomb 80,27, shallow skyphos, Early Geometric II–Middle Geometric I; Pyre 21?,5, cup, Early Geometric II–Middle Geometric I; Pyre 23c, Subprotogeometric IIIa; Pyre 33, skyphos, Early Geometric II) [42]. It is worth stressing that the motive of the tight-packed multiple zigzag was introduced for the first time in the Early Geometric II phase in Athens suggesting that an earlier date for the sherd from Sidon is not possible [43].

**Fig 29:**
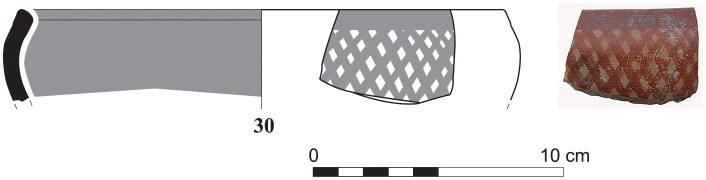
Fragment of a Greek shallow bowl from Sidon phase F dating to the Middle Geometric I / Subprotogeometric IIIA.

Another sherd from a shallow bowl (S/110544/8357) of Euboean fabric with cross-hatching on the handle-zone was found directly on the floor of Room 1/3 of Phase F (Fig 29). This decoration, which was quite uncommon for shallow bowls, was mainly used in the earlier phases of the Subprotogeometric period. The vase from Sidon may thus be dated at the latest to Middle Geometric I, as has already been argued [14].

**Fig 30:**
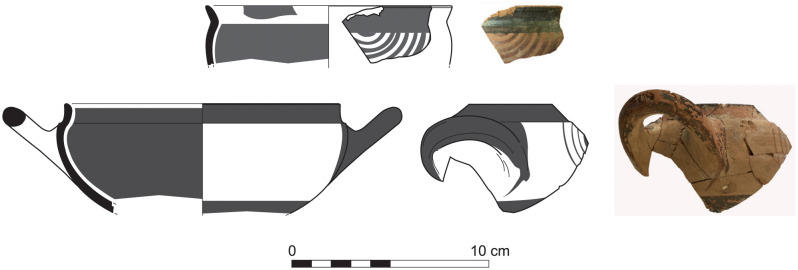
Fragments of Euboean (NAA) pendent semicircle skyphoi from Sidon phase I dating to Middle Geometric II / Subprotogeometric IIIB.

**Fig 31:**
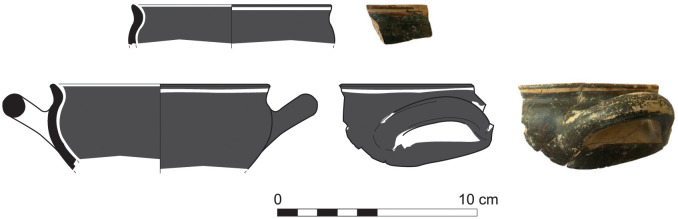
Fragments of Greek (NAA) monochrome skyphoi from Sidon phase I dating to Middle Geometric II / Subprotogeometric IIIB.

More sherds of Greek Geometric pottery came from well stratified contexts of Phase I at Sidon lying directly on the floors of the main building’s rooms 1 and 3 excavated at the College Site. These finds comprise two pendent semicircle skyphoi of type 4 (S/7555/8315) and 5 (S/7220/8290) (Fig 30), as well as two monochrome skyphoi (S/7218/8271 and S/7495/8304) (Fig 31), all of them dating very well to the Middle Geometric II period [14, 41]. All these four sherds, together with 14 other fragments from Greek Geometric vessels, have been analysed with Neutron Activation by Hans Mommsen. All but two sherds proved to belong to the best defined Euboean geochemical group EuA. The monochrome skyphos S/7218/8271 showed a different origin since it was grouped in a cluster probably originating from western Peloponnese and another sample was defined as geochemical singleton (Gimatzidis and Mommsen forthcoming).

**Fig 32:**
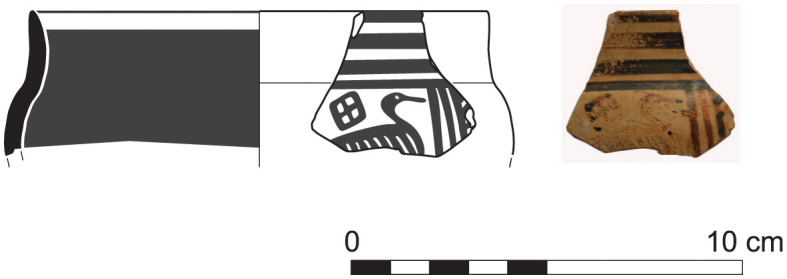
Fragment of Euboean bird skyphos from Sidon phase K dating to Late Geometric I.

**Fig 33:**
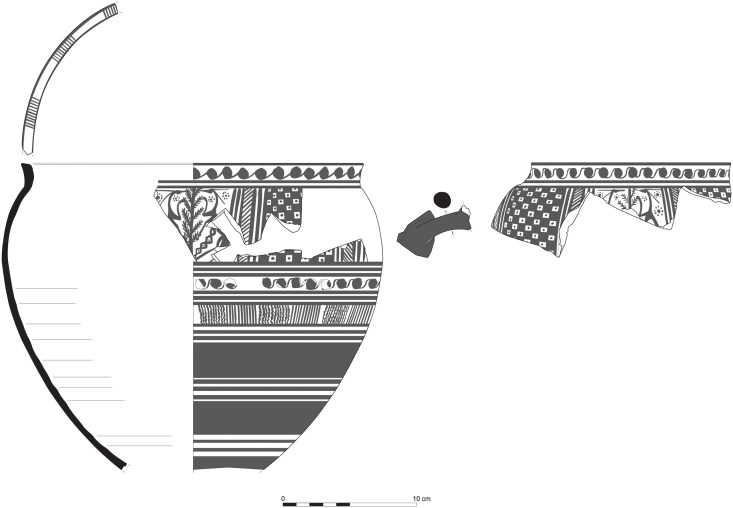
Euboean (NAA) crater from Sidon dating to Late Geometric I.

The long stratigraphic sequence at Sidon extends with Phase K into the Late Geometric I period. The first clearly Late Geometric pottery sherd was found in a well-defined context directly on the floor of room 2 of this phase. A skyphos of Euboean origin with high and straight rim and a bird in a metope in the handle zone (S/7529/9211) is a pottery type dating to Late Geometric I (Fig 32). The most emblematic Greek Geometric vase that was found at Sidon also relates to contexts dating to some of the latest occupation phases: this is a crater of Euboean origin (as shown by Neutron Activation Analysis), that is was decorated with the Tree of Life flanked by two rampant goats (Fig 33) (the bird skyphos, which was tentatively associated with phase J in a preliminary report, now has its definite provenance in phase K; less straightforward is the attribution of the crater to some particular phase [14]). The introduction of this motive to the Euboean iconography is usually attributed to the so-called ‘Cesnola Painter’, whose work was also dated to the Late Geometric I [41].

The rest of the Greek pottery assemblage comprises some dozens of fragments of pendent semicircle plates and skyphoi, as well as a few other types, all of them dating to the same time span extending from the Late Protogeometric/Early Subprotogeometric to the Late Geometric I period. Although some of these sherds can be related in one or the other way with some building phase at Sidon, we prefer not to consider them in this paper, where we put focus on sherds (as discussed above) that come from best-defined, primary contexts.

The correlation of the Greek pottery with other ceramic wares at Sidon as well as the 37 ^14^C-AMS-dates that were obtained on short-lived material–mainly olive pits and few bones–has important implications for the reconstruction and synchronisation of the many different Mediterranean Iron Age chronological systems. In the first place it is worth stressing that none of the 36 radiocarbon ^14^C-ages that were obtained on material from Phases A to I have dates after 900 calBC (a single analyzed sample came from Phase K). The majority of our ^14^C-ages (N = 23) come from Phases C and D. Following exclusion of the outliers (N = 8), these ^14^C-ages provide dates of 1144–1074 calBC and 1074–1002 calBC for the two Phases, respectively. All of the new Sindos dates are consistent with the revised Aegean chronology, as achieved at Sindos, where Early Geometric was raised up in the second half of the 11^th^ century calBC (Phase 10 at Sindos) [6]. The bowl with multiple zigzag (Fig 28; S/8569/8579) that comes from a secure context of Phase D was dated to the Early Geometric period, which is the highest possible relative date for this sherd. Finally, three ^14^C-ages that were obtained from secure contexts of phase I, which was pottery-dated to Middle Geometric II, suggest an absolute date for this phase in the second half of the 10^th^ century calBC. Again, this agrees well with the calibrated ^14^C-ages derived for the Middle Geometric II strata at Sindos (Phase 8) [6].

#### Methods and approaches to the use of Greek pottery for chronological purposes in the eastern Mediterranean

Greek Geometric pottery finds in the eastern Mediterranean have been commonly suffering overinterpretation on both cultural and chronological terms. On the one hand, they were regarded as indicators of the ethnic origin of their carriers and consumers, or were perceived as gifts exchanged among elite groups through a consumption-focused approach that was coined by anthropology during the last two decades of the previous century. On the other hand, early Greek pottery finds were treated as chronological markers that could be effectively used to correlate the Aegean and eastern Mediterranean chronologies, thus providing a framework for the construction of a broader Mediterranean chronological system. It has often been stated that the use of the Greek pottery as a tool for the construction of a supraregional chronological system is due to its fine forms and elaborate decoration types, that already now support an analytical ceramic typology at high-dating resolution, and would probably even support a quantitative pottery seriation (although this has not yet been attempted). It is nevertheless useful to stress again–and in particular when discussing the potential application of pottery seriation by Correspondence Analysis–that the Greek absolute chronology was gained in the past by means of usually very few, not well stratified and also not precisely datable Geometric sherds in the southern Levant. The temporal definition of significant phases of the Greek Geometric period was attempted long time ago by means of a handful of Greek sherds from destruction layers at sites such as Tel Abu Hawam, Megiddo and Samaria that could allegedly be dated, usually through their correlation with some historically documented invasion [44]. In the meantime, the historical dating of these sites has been put in question, repeatedly, and the relative dating of the Greek pottery types that were recovered has also been revised, several times. Yet, these challenges have apparently only barely affected the conventional chronological schema of the Greek Geometric period that was established by Nicolas Coldstream in the 1960’s [14]. The conventional chronological system is instead vigorously defended, if only to maintain consistency in interdisciplinary studies, for convenience in material studies, and in certain cases for purposes of ideology.

Many efforts have been undertaken during the last two decades to provide further support for the conventional Greek Early Iron Age chronology, especially after it was first challenged at certain sites in the Aegean [4] and western Mediterranean [1, 2, 5] by means of radiocarbon and dendrochronological data. Nevertheless, although the new chronological evidence based on radiocarbon dates has at least partly replaced the historical arguments–and these are now close to exhaustion in the southern Levant–the archaeological record in the eastern Mediterranean has itself not been further significantly advanced. It is still the case that single or sparse pottery finds that cannot be precisely dated, and which usually come from insecure contexts, are used to argue for the correlation of the Greek and eastern Mediterranean chronology, albeit by means of supposedly combined historical and scientific evidence [14].

#### The chronological implications of the Greek Geometric pottery from Tell es-Safi, Megiddo and Tel Rehov in the southern Levant

The discussion over a single sherd from a skyphos with a wavy line in the reserved handle zone from Tell es-Safi, the Biblical city of Gath, which comes neither from a primary context nor can it be dated with precision to any one or the other Aegean period, represents a case of overinterpretation and imprecision in the synchronisation of regional Mediterranean chronological systems [45]. Previous critique renders the radiocarbon data from that site effectively useless, at least for purposes of chronological correlation between the Aegean and the eastern Mediterranean [14].

**Fig 34:**
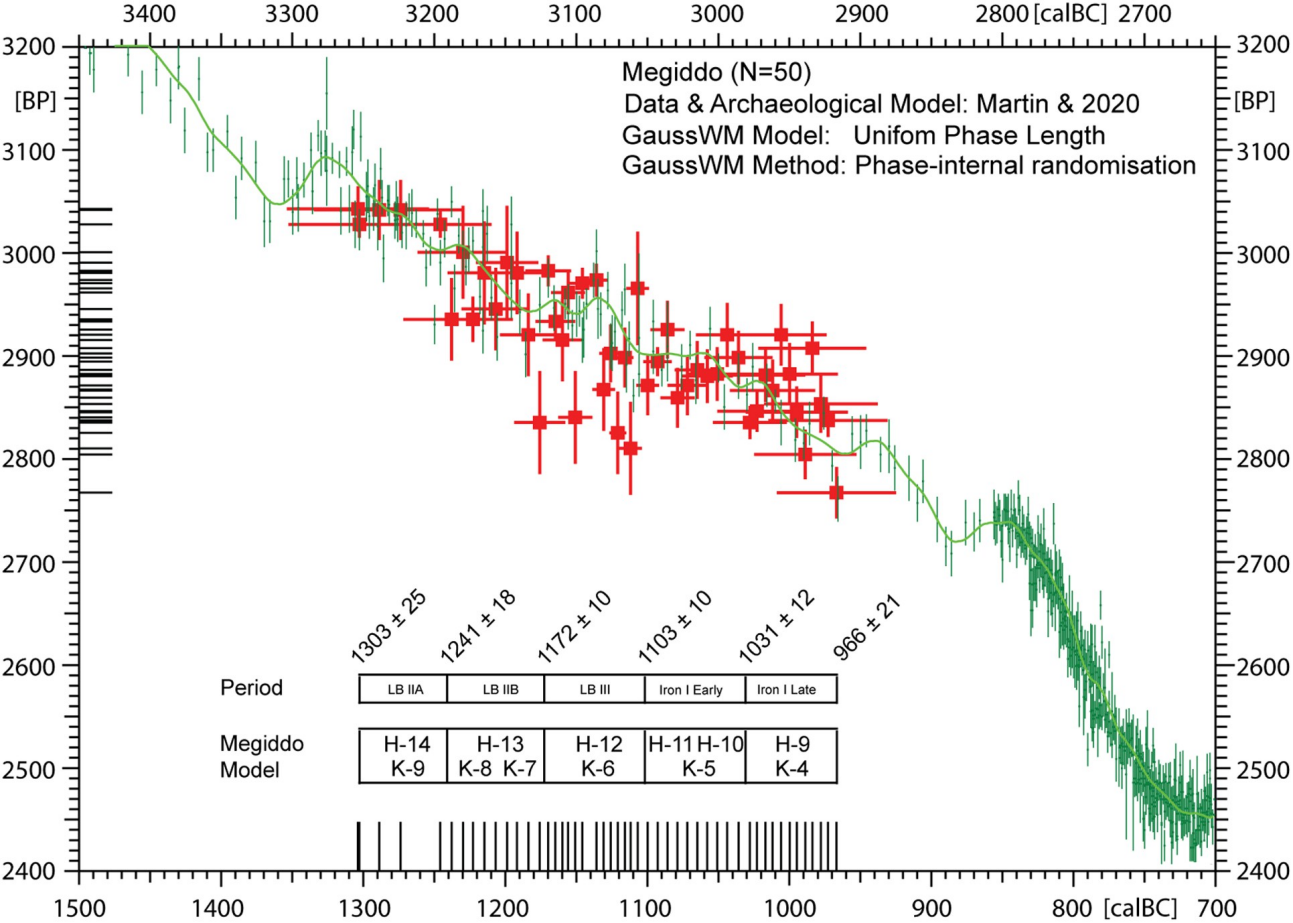
GaussWM-derived chronology for Megiddo based on the same ^14^C-data (N = 50) and archaeological age-model as published by [40].

Similarly overinterpreted are some new finds from Megiddo, the radiocarbon chronology of which is shown in Fig 34. These finds have been misleadingly presented as evidence supporting the conventional chronology [46, 47]. In fact, these comprise four monochrome pottery fragments that do not allow for stylistic dating, and a single sherd of a pendent semicircle plate. The latter is one of the less well dated Greek Geometric pottery types, whose chronological sequence has apparently been misunderstood at Megiddo. This pottery type that was common throughout the Subprotogeometric period appears for the first time in contexts dating between Late Protogeometric and Early Subprotogeometric I in the Aegean, where it was mainly produced. Numerous finds from Cyprus, Cilicia and Cyprus show that its circulation increased during the Subprotogeometric III/Middle Geometric period [14]. Finally, we still miss evidence for the understanding of this shape’s typological sequence, which means that the relative dating of such finds within the large chronological span of its period of production is either not possible or can be done only with the reservations mentioned above [41]. Despite all these caveats, the fragment of the pendent semicircle plate from Megiddo was thought in a circular reasoning to date to the Late Protogeometric period because in this way it could support the expected Megiddo-based synchronisation of the Greek ceramic chronology with the locally obtained radiocarbon evidence and consequently also the conventional Aegean chronology [46]. This reasoning followed a misperception of the pendent semicircle plate as a pottery type allegedly dating between Late Protogeometric and Subprotogeometric II, although its main period of production and use dated from the end of Late Protogeometric to the Subprotogeometric IIIb period. The plate type from Megiddo could in fact be dated to the Subprotogeometric III/Middle Geometric period as some parallels suggest. As already stressed, this was the period during which the pendent semicircle plate was more commonly used in the eastern Mediterranean [14, 41, 44].

**Fig 35:**
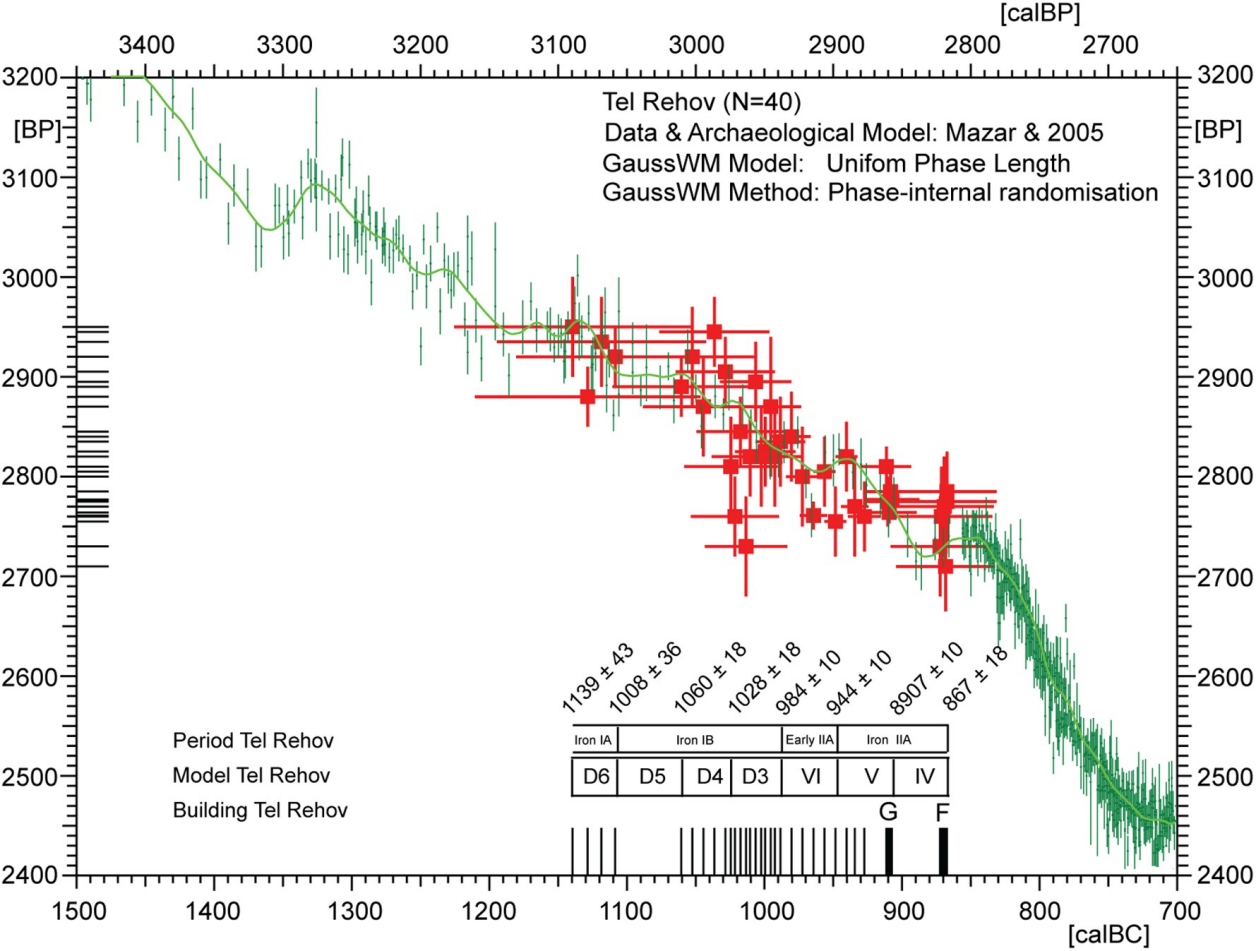
GaussWM-chronology for Tel Rehov based on the same ^14^C-data (N = 50) as published by [50], and using an equivalent archaeological age-model.

More value for chronological correlations than the previous finds from Tell es-Safi and Megiddo has a small assemblage of sherds from 14 Greek Geometric vessels that was recovered a few decades ago at Tel Rehov. The radiocarbon chronology of Tel Rehov is shown in Fig 35, along with a reminder (as noted above) that the stratigraphic age models achieved by application of Bayesian Sequencing [50] and by GaussWM (this paper) are practically identical.

These ceramics have been published in several papers not always supporting the conventional chronology as originally expected. Half of the sherds from Tel Rehov were presented in a first study by Nicolas Coldstream that was supplemented in the following years by new finds illustrated in further reports [48–50]. Unfortunately, only very few of these sherds can be considered useful for purposes of chronological correlation since most of them are neither well preserved, nor reliably datable using traditional typological approaches. Nevertheless, a couple of sherds that were presented as belonging to the same skyphos from Stratum IV, and several sherds of a pyxis that were found in five different loci belonging to different layers and probably also settlement phases, do deserve some further attention [48, 50].

The sherds of the pyxis were not found in a primary context but scattered over a long distance on the slope of the mound that was eroded by a gully. The context of this vase cannot be thus considered as a secure starting point for any discussion about chronology. Even if we accept the suggestion of the excavator that it was originally used in Stratum V ‘the SPG (Subprotogeometric) II-IIIa Euboean pyxis poses a problem’ for the conventional chronology, as Coldsteam and Mazar initially admitted, since ‘it would predate the date suggested by Coldstream by some 50 years’ [48]. It was again rather through circular reasoning than based on solid archaeological arguments that this pyxis was recently dated with alleged precision to Subprotogeometric II, in which case its correlation with the available radiocarbon evidence better fits the conventional chronology [49, 50]. Stratum V of Tel Rehov is chronologically placed between Phases 9 and 8 of Sindos that were securely dated to Middle Geometric I and II respectively [51]. The pyxis cannot support the conventional Aegean/Mediterranean chronology, even if it is relatively dated with the suggested unusual precision to Subprotogeometric II. Its dating instead to Subprotogeometric IIIa / Middle Geometric I would be fully consistent with the revised Greek chronology as both ^14^C-ages and historical evidence from Tel Rehov imply. In that case, the data from Tel Rehov would fully agree with those from Sindos placing the transition from Middle Geometric I to II within the second half of the 10^th^ century calBC [6].

Two other sherds from Stratum IV of Tel Rehov have been taken to belong to the same skyphos decorated with multiple zigzag and were dated by Coldstream to the early Middle Geometric I period [48]. As in the case of the pyxis discussed above the skyphos was also dated with an uncommon precision to the beginning of Middle Geometric I. Again, this approach that does not depend on any statistical or typological evidence serves in fact to provide this piece with a date closer to the conventional Greek chronology. Otherwise the latter would be contradicted by the radiocarbon dates available from Stratum IV at Tel Rehov, as Amihai Mazar in this case actually mentions [52]. Our modelled dates from Tel Rehov place Stratum IV into the first half of the 9^th^ century calBC significantly deviating from the conventional chronology, according to which Middle Geometric I should date between 850 and 800 histBC (Fig 35).

In the most recent re-publication of the Greek finds of Tel Rehov there is, however, some additional information about these sherds that did not join and were found in ‘two small adjacent chambers of Building CF’ [50]. We understand thus that the two sherds may well belong to two different vessels no matter how similar their fabric may be since skyphoi of this kind show an impressive standardization in their technology. On the one hand, the rim fragment form Tel Rehov Stratum IV comes from the monochrome part of a skyphos whose main decoration is not preserved. On the other hand, the body fragment with the multiple zigzag may come from any Attic/Atticizing skyphos dating from the Early Geometric II to Middle Geometric II, when this motif was in use in Attica and Euboea. Given these ambiguities it makes more sense to attempt to date the best datable rim sherd separately.

The drawn reconstruction of the skyphos, from which the rim sherd comes and which was presented in previous reports, shows a deep bowl with a not particularly low but offset flaring lip slightly overhanging the marked shoulder [48–50]. Skyphoi with such typological features appear in fact only as late as the Middle Geometric II period and are certainly not common among Middle Geometric I ceramic assemblages in Attic or Euboean necropolises; skyphoi dating to Middle Geometric I are usually shallower and with shorter rims [48, 49]. A comparison with better dated and stratified finds in the Aegean shows instead that the profile of the skyphos from Tel Rehov finds its best parallels among Euboean skyphoi with variable decoration (cat. nos 164, 167, 169, 176) from Phase 7 of Sindos that was securely dated to Late Geometric Ia [51]. We suggest thus the dating of the skyphos from Tel Rehov between Middle Geometric II and Late Geometric Ia. If this vase was indeed decorated with multiple zigzag, it can be taken as a developed version of a zigzag skyphos, which stopped being produced only after Middle Geometric II. No matter how the skyphos from Stratum IV of Tel Rehov is dated (Middle Geometric I or more probably Middle Geometric II), the radiocarbon dates from the same settlement phase do not support the conventional chronology. The dating of Stratum V to Subprotogeometric IIIa/Middle Geometric I and Stratum IV to Subprotogeometric IIIb/Middle Geometric II are instead consistent with the revised Aegean chronology suggested at Sindos [6].

Any attempt for the relative dating of the other Greek pottery sherds from Tel Rehov is even less reliable than that of the aforementioned pyxis and skyphos [44]. A small wall pendent semicircle skyphos fragment (no 4) that was dated already in the first report on the Greek pottery from Tel Rehov between Subprotogeometric I and IIIa (Early Geometric I and Middle Geometric I) can in fact belong to any pendent semicircle skyphos type dating from the Late Protogeometric to Late Geometric Ia period [48]. In two most recent papers [49, 50], where a few new finds were additionally presented, a monochrome skyphos body fragment (no 8) that could date to any phase of the Early Iron Age was precisely assigned to Subprotogeometric II/Early Geometric II, and two rim sherds from a skyphos (no 9) and a crater (no 10) with again lacking precise pottery-date were ascribed to the same period (Subprotogeometric II). Similarly unrealistic is the dating of a lower wall and base fragment of a bowl to the Late (!) Middle Geometric period (no 14). Equally ambiguous was also the attribution of two rim sherds from bowls to Middle Geometric (nos 12 and 13).

#### Greek pottery and the new radiocarbon dates from Tell Tayinat in the Amuq plain, northern Levant

A large dataset of 49 radiocarbon determinations has recently become available from Tell Tayinat, a major site in the Orontes valley, where large amounts of Greek Geometric pottery were consumed [28]. In Fig 9 (see above) we provide a detailed comparison of the chronological results achieved by age-modelling using OxCal and CalPal. Looking beyond the details, what we judge important is that we have independently confirmed the results of Manning et al. 2020 [28]. Remaining differences (decadel-scale) are covered by the modelling errors, as well as by differences in probabilistic terminology between OxCal and CalPal. The main difference, perhaps more important, is that in the OxCal-application [18] the date of 738 calBC for the Assyrian conquest of Tayinat has to be entered as an (error-free) prior (namely to stop the sequence from sliding far into Pearson’s Peril), whereas in the CalPal-model effectively the same date (730 ± 20 calBC) is model-generated.

In addition to the new ^14^C-data, there is a large and steadily growing assemblage of Greek pottery (mainly drinking vessels) from Tel Tayinat, that is the capital site of the Patina kingdom. More than a decade ago 86 sherds of Greek Geometric Pottery, found during the older excavations of the Oriental Institute of the University of Chicago during the 1930s as well as from the more recent Tayinat Archaeological Project, have been registered at Tell Tayinat [53]. The dominant type was the pendent semicircle skyphos comprising 80% of the total Greek Geometric pottery assemblage. Unfortunately, the exact stratigraphic correlation of the recent ^14^C-determinations with the Greek pottery finds at Tayinat, particularly those from the older excavations, is hampered by deficient stratigraphic documentation during these earlier field works [53].

The information about the Iron Age Greek pottery from Tayinat is meagre. Nevertheless, it does seem clear that the contextualization of the Greek wares at Tayinat presents certain chronological inconsistencies. Pendent semicircle skyphoi have been documented as having been used in contexts of Building I throughout its Building Periods (BP) 2 and 3. The latter BP 3 was dated by means of other finds to the final third of the 8^th^ and to the early 7^th^ century BC [53]. If the ascription of the use of pendent semicircle skyphoi to this Period is not due to a stratigraphic deficit, it certainly represents a chronological misperception of BP 3. According to recent secure stratigraphic data from several sites in the Aegean and Mediterranean [51], pendent semicircle skyphoi cannot be dated after Late Geometric Ia. Additionally, this Greek period can by no means be dated to the last third of the 8^th^ century BC or later.

The modelled radiocarbon dataset from Tell Tayinat was recently interpreted in support of the conventional chronology [28]. In the first place, the earliest use of ‘LH IIIC-style ceramics’ was attested in layers of Field Phase (FP) 6c that was ^14^C-dated to the 12^th^ century calBC. This occurrence of ‘LH IIIC-style pottery’ was taken to contradict the data presented at Assiros in the northern Aegean that suggested that the transition from the Late Bronze Age to the Early Iron Age dates to the 12^th^ century [28]. Nevertheless, the data from Assiros suggest that pottery of Mycenaean type and origin was still used during the 12^th^ century in the Aegean, allowing the adaption of this ceramic style during the same period in the eastern Mediterranean [4]. This is, however, not the only or even the most significant misunderstanding of Aegean ceramic chronology and its mode of diffusion, in particular, when such issues are treated in non-Aegean cultural contexts. What is interpreted as ‘LH IIIC-style’ pottery both at Tayinat, and at other sites in the northern Levant, in fact shows features that indeed appeared for the first time during the later phases of the Aegean Late Bronze Age, but continued to be in use also during the following Protogeometric period. In other words, the term ‘LH IIIC-style’ ceramics is in fact used to describe pottery bowls with S-profile that were produced as late as the Late Protogeometric in the Aegean and were often decorated with zigzags, tassels and other linear motifs of Mycenaean origin. Finally, the inner decoration of the ‘LH IIIC-style’ bowls may reflect ‘Mycenaean’ traditions as well as local adaptions and tastes in the eastern Mediterranean. Having said that we may now understand why the first appearance of this pottery style during FP 6c at Tayinat was ‘extremely rare’, while it began to increase only from the next FP 6b onwards and continued to be used until FP 3 [54]. Not unexpectedly, then, the modelled radiocarbon dates from Tel Tayinat show that FP 6c–a and 5a–b extend over a considerable span of time, that is from the 12^th^ through the 11^th^, all the way through to the first half of the 10^th^ century calBC, when the ‘LH IIIC-style’ pottery was already outmoded in the Aegean. In combination of the archaeological and analytical data, what we see at Tayinat is that this pottery initially reflects both the older, as well as the current Aegean ceramic types, and then finally represents the emergence of a local idiosyncratic ceramic style. The ‘LH IIIC-style’ pottery from Tayinat would have been typo-chronologically better defined, if there were analytical data–obtained by means of Neutron Activations Analysis–available about its origin.

At this point it is significant to stress that there is only one radiocarbon-analyzed sample available for the FP 6c [28], whose contexts are far from secure due to large pits and silos that were opened at the site [55]. Additionally, both this single date from FP 6c as well as all nine dates from the younger FP 6a and b were measured on wood-charcoal. Thus, even if we accept a date before 1100 calBC for FP 6c, it is still not possible to state *precisely* when this phase dates. The pottery of ‘LH IIIC-style’ became popular only during the next phases dating to the 11^th^ and 10^th^ centuries calBC at Tayinat, i.e. when its motifs were becoming outmoded even in the Aegean Protogeometric pottery styles. It seems though that there was in fact not any ‘LH IIIC-style’ pottery at Tayinat in the late 2^nd^ and early 1^st^ century BC, but rather a ceramic category of an early Aegeanizing style selectively using forms and decorative motives that were common during the Late Helladic IIIC as well as the Protogeometric period, and which continued to be used in later periods in the eastern Mediterranean. The first appearance of the new Aegeanizing style in the 12^th^ century calBC at Tayinat, and that was continued in the next centuries, does not present the requested analytically precise and archaeologically secure chronological evidence, neither on-site, nor in terms of the Aegean periodization. Instead, the pottery chronology at Tel Tayinat represents the beginning and continuation of some long processes of ceramic style adaption, and style-selection, but which do not depend entirely on these older and geographically remote ceramic traditions.

Turning now to the more recent excavations, the presently scarce information about the few recent Greek pottery finds at Tayinat is also not particularly helpful for the archaeological evaluation of the newly obtained radiocarbon dates. The small rim fragment that was recovered in a context of Field Phase 2b (and probably also the later FP 2a?) above a cobblestone paving in the surroundings of Building XVI (Field 2) may belong to a pendent semicircle skyphos or some other similar ceramic shape with different decoration. The pottery assemblage above this cobblestone surface did not form a primary context but rather contained material from several chronological phases (Iron Age I and II) and was thus dated initially from the late 9^th^ to early 7^th^ century BC following the conventional chronological system [56].

Two other better preserved pendent semicircle skyphoi of type 5 were found in contexts of FP 2 that comprised part of a large courtyard building (Field 5) thought to have been of Assyrian type and hence origin, i.e. dated after 738 BC. Unfortunately, it is not clear from the preliminary reports whether the pendent semicircle skyphoi were used during the first (PF 2d) or second (FP 2b) occupation phase of the building. Nevertheless, the whole assemblage was dated to the late 8^th^– 7^th^ century BC that is the Neo-Assyrian phase of the site (Iron Age III) [56]. Again, these dates certainly do not fit either the conventional or revised Aegean chronology, and are also not consistent with the established seriations of the pendent semicircle skyphoi [51].

**Fig 36:**
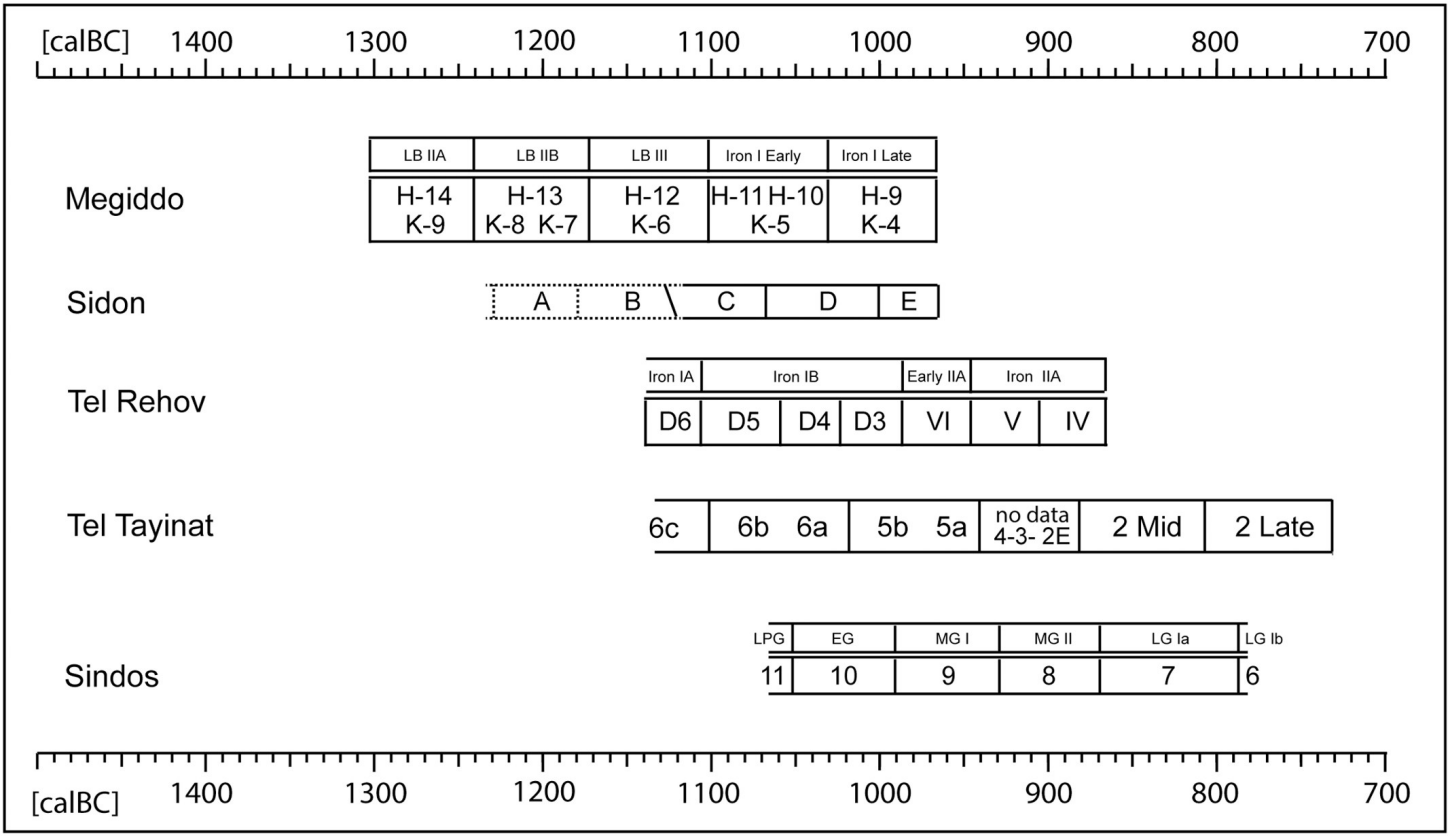
Comparison of the ^14^C-chronologies for the study sites, based on GaussWM.

Provided that at least some of the subphases of FP 2 in Field 5 correlate with those of FP 2 in Field 2, as the authors imply, the chronology obtained on some olive pits would suggest a higher date. PF 2b (Phase 2 Late 2) was radiocarbon dated between 793 and 733 calBC [28]. It is still not clear whether PF 2d of Field 5 correlates with an earlier building phase in Field 2, which would be the expected find context for the pendent semicircle skyphoi. Nevertheless, it would be more reasonable to wait for the detailed publication of the Greek pottery and their contexts from Tell Tayinat before drawing any conclusions about the consistency of the local stratigraphic sequence and recently obtained radiocarbon dates. For the moment, based on currently available data from the eastern Mediterranean, we can describe the combined pottery- and ^14^C-based chronology at Tayinat as consistent with the revised Aegean chronology, as previously suggested at Sindos in the northern Aegean [6], and which is now further supported by our new data from Sidon (Fig 36).

### The Cypriot pottery context

#### The implications of Gjerstad’s Cypriote chrono-typological system

To understand the potential chronological significance of the Cypriote Iron Age pottery in Sidon it is of importance to briefly discuss some of the challenges of its classification. This is to avoid misunderstanding and partly also misuse of this group of finds. Indispensably, this directs us to Einar Gjerstad’s famous classification of the island’s Iron Age pottery [57–59]. Due to the absence of more recent alternatives, this is still the most influential, if not to say the only possible reference [60–63]. Due to this pigeonhole restriction and its entailments regarding the finds from Sidon, it goes without further saying that the weaknesses and concomitant consequences of Gjerstad’s classification need to be illuminated in more detail.

Two studies can be considered as the premises of Gjerstad’s monumental research: One goes back to the 19^th^ century, when Thomas B. Sandwith has developed a relative chronology by grouping a selection of tomb inventories [64], while the other one comprises the efforts by John L. Myres and Max Ohnefalsch-Richter to categorize the Iron Age pottery in rather general ‘technological groups’ [65, 66]. The latter was guiding Gjerstad’s division of the pottery in what he called wares. By this he meant categories of rather general technical executions which he named White Painted, Black Slip, Red-on-Black, etc. Geared by the shapes and decoration of his type fossil White Painted pottery he split the material in typological groups [63, 67, 68]. His establishment of the groups’s chronological sequence was based on 1223 vessels [59] of which some two thirds originated from tombs. Similar to his above mentioned forerunner Sandwith, Gjerstad was also confronted with tombs which usually involved multiple burials of different time periods [69–71]. Their stratigraphical differentiation was and today still is difficult and thus has often been misinterpreted as a single burial layer [60]. The methodological inaccuracy of this pseudo-seriation still causes many difficulties in the (at first glance) seemingly elaborate typo-chronology. In particular, as noted by Maria Iacovou, this find-situation has quite often led to a ‘compression of wares’ which raises serious doubts that an affiliation of single types to particular phases is indeed possible, to the extent Gjerstad suggested [60, 67, 72–74].

Similarly critical, Joanna S. Smith assumes that only about 8% of Gjerstad’s material stems from stratified contexts in settlements or sanctuaries [63]. Until today, the few stratified settlement contexts for the so-called Cypro-Geometric period still hampers research on Cypriote chronology. Gjerstad used these contexts to somehow verify the relative sequence of his typological ‘phases’ [59]. Beyond the fact that it is difficult to trace his method in detail, there is also the problem that Gjerstad considered the pottery assemblages of mortuary, sanctuary and settlement context to be directly interchangeable [59]. Louise Steel and later Stella Diakou question this approach arguing for an at least partly specialized production of fine wares for funeral rites [60, 75]. This mixing of the different functional contexts within Gjerstad’s chronological frame implicates yet further insecurities. A similar problem exists due to the fact that neither detailed technical aspects [63, 67, 73] nor regional differences were integrated [73]; this field has only recently been more systematically investigated by Anna Georgiou [70, 76, 77].

To convey his relative sequence into absolute chronological dates, Gjerstad attempted to relate the Cypriote pottery to the Levantine sequence by means of cross-dating [59]. It is expectable that intricacies as for instance particular shape choices or regional preference did not attract his attention at this stage of research. However, it became quickly clear that his cross-dating has been based on problematic contexts [74, 78], or on the highly debated chronological frameworks of the southern Levante [79]. Consequently, the limits and subdivisions of the Cypro-geometric period are insecure [76, 80], and in particular the absolute dates of its subdivisions (Cypro-Geometric I: 1050–950 BC, Cypro-Geometric II: 950–850 BC, Cypro-Geometric III: 850–750 BC, [59] have to be handled very cautiously.

Due to the described methodological weaknesses of Gjerstad’s system it is evident that assignments of few or even single sherds to his typo-chronological phases are not only inadvisable (as also applies to other systems), but are actually misleading at the current state of Cypriote Iron Age pottery classification. Gjerstad’s types can actually run much longer than is often assumed, and in consequence even larger assemblages can only be used to provide some general hint at how to clarify the chronological issues of the Cypriote pottery, and its relation to the Levantine sequence. At best we can use his system to typologically indicate what we are discussing and perhaps provide an educated guess regarding a relative-chronological sequence. Additionally, we have to keep in mind that certain shapes and decorations might have been produced for different time spans depending on region or the intention of their use. Pinpointing absolute dates with Cypriote pottery is surely beyond the scope of present research. Moreover, keeping in mind the dependence of the Cypriote absolute dates on the chronologies of the Levante, it is obvious that it leads to a circular argument when one tries to date Levantine site through Cypriote pottery, as is sometimes attempted [62]. In the end only detailed excavation and study of settlement stratigraphies on Cyprus itself can help to resolve this dilemma and significantly bring forward research on the chronological assignment of Iron Age pottery. Notwithstanding the need for research on Cyprus itself, the material from Sidon provides the chance to address some interesting questions in the field of chronology.

#### The Cypriote ceramic material from Sidon

A minimum of 37, mostly highly fragmented vessels of Cypriote origin or manufacture with Cypriote-style affinities have been found in Sidon Phases A to G [13]. In terms of architecture, these phases can be split into two groups: the group of earlier Phases (A-D) is separated from the group of later phases (F-G) by a radical change in the internal structure of the major building.

From Phase A, the shoulder fragment of an amphoriskos decorated by alternating black and brown bands can be potentially identified as belonging to the Proto-White-Painted/Proto-Bichrome group of LC IIIB. This shape appears for the first time in the repertory of the Proto-White-Painted (PWP) pottery [13, 81, 82], but is also used in the following phases. The pottery category has not yet been securely identified outside Cyprus [72, 83, 84] due to the fact that an assignment especially of smaller fragments to PWP (and not to Cypriote Geometric) is mostly based on its painting [72]. Thus, all such identifications should be treated with caution.

**Fig 37:**
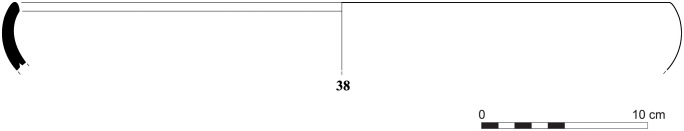
Rim fragment of a shallow Plain-White bowl (125693/4510).

**Fig 38:**
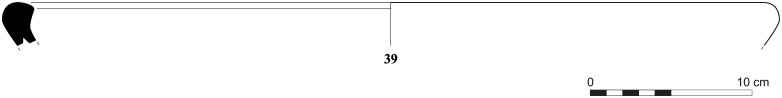
Rim fragment of a shallow Plain-White bowl (125697/4510).

**Fig 39:**
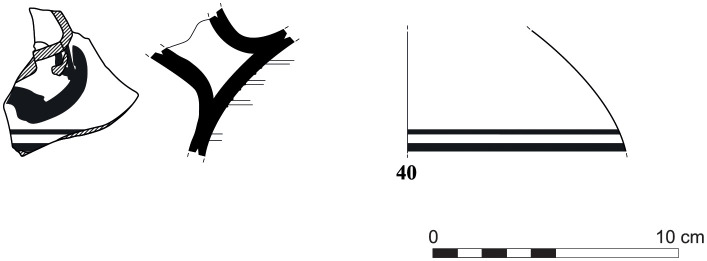
Body and spout fragment of a White Painted I-II strainer jug (125772).

The six fragments of vessels–three shallow Plain-White I-II bowls (Figs 37 and 38) [13, 59, 60, 81], body sherds and a spout fragment of a White-Painted I-II strainer jug (Fig 39) [13, 59] and the body of a Bichrome I-II jug—originate from rooms 6, 8 and 9 in Phase B. In the light of Joanna Smith’s consideration that CG I and II represent contemporaneous ceramic styles, it is not very surprising that both typological groups occur in the same chronological horizon of Sidon [63]. Similar observations have been for instance also made for the stratigraphical sequence in Tel Dor [8].

**Fig 40:**
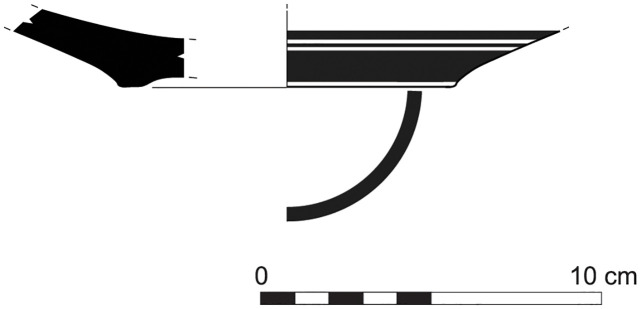
Bottom fragment of a shallow bowl, White Painted II (121646/4469).

Only one fragment which could be only generally assigned to White-Painted pottery originates from Phase C that dates from 1144 to 1074 calBC [13]. Meanwhile, the more or less contemporary Subphase C1 has brought to light a minimal number of three vessels: one originates from Room 6 and could be generally categorized as White Painted; another can be assigned to Gjerstad’s chrono-typological group II [13] (Fig 40); the third was found on a floor surface outside the room. Due to its small size it can again only generally be identified as White-Painted [13].

**Fig 41:**
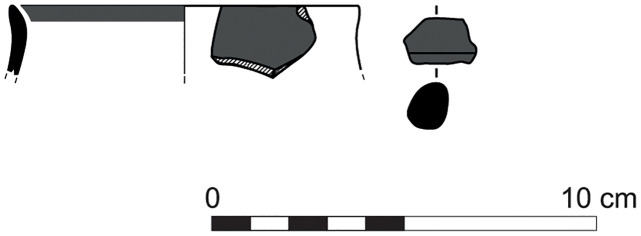
Rim and handle fragment of a White Painted I deep bowl (118109+ 118110).

Two fragments of a deep bowl with horizontal handles from Phase D, which dates from 1074 to 1002 calBC were classified as either PWP or more likely White-Painted I-II (Fig 41) [13]. Subphase D1 yielded two further White Painted fragments of a closed and open shape, but only the cross-shaped pattern of the open vessel (probably a deep bowl) broadly indicate its belonging to White Painted I-III [13, 59].

**Fig 42:**
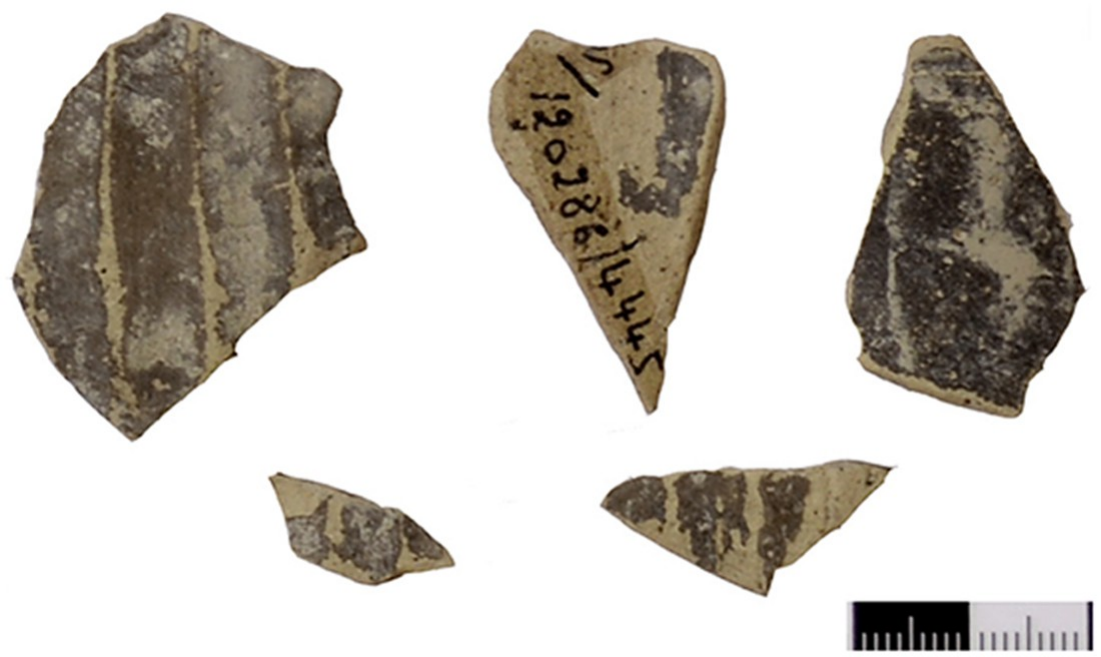
Body sherds of a juglet, Black Slip (S/120286/4445).

**Fig 43:**
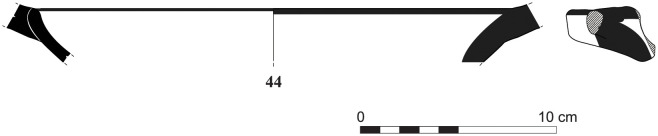
Rim fragment of a shallow bowl White Painted II (120572/4446).

With Phase E the internal structure of the building significantly changes together with the gradual emergence of a small quantity of Phoenician Bichrome Ware which then flourishes in Phases F and G [12]. Pottery fragments of three Cypriot bowls and three small Cypriot containers were discovered in this phase, all of which belong to Gjerstad’s chrono-typological group II and possibly III [13]. Among them there are five fragments of a Black Slip jug from Room 6 (Fig 42) with carefully made fluting which could be an indication for its assignment to Gjerstad’s types I or II [13], found along with two shallow White Painted II bowls [13, 60]. A body sherd of a closed vessel has parallels to a decoration scheme which has been assigned by Gjerstad to Bichrome III (Fig 43) [13, 59].

**Fig 44:**
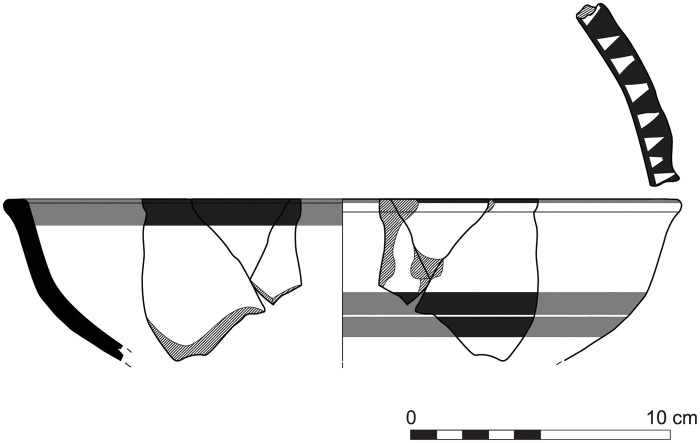
Rim fragment of a shallow bowl, White Painted IV (122409/4483).

**Fig 45:**
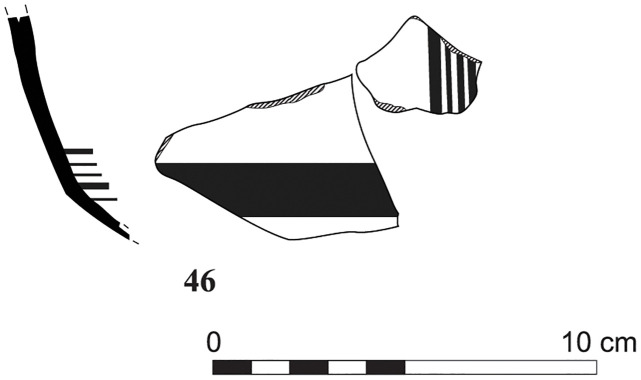
Two body sherds of a deep bowl, White Painted III (97731/97732/9116).

Further indications for the occurrence at Sidon of Gjerstad’s later types (III and IV) continue during the transitional Phase E/F, which produced the lower part of a shallow Bichrome III/IV bowl from Room 7 [13, 73, 85]. In Phase F bowls and jugs of Gjerstad’s chrono-typological Phases III and IV dominate: a shallow White-Painted IV bowl (Fig 44) [13, 86], a White-Painted III bowl [13, 87] or a White-Painted III jug [13, 59] (Fig 45).

**Fig 46:**
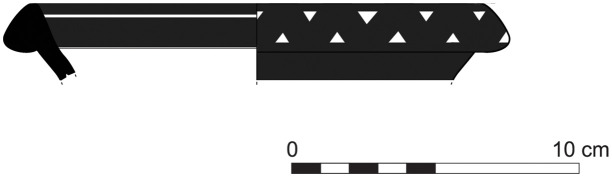
Rim and body fragment of an amphora, White Painted IV (109478+109480/8324).

Similar types of pottery were found in Phase G, but with now increasing amounts [13]. In this phase Cypriote Black-on-Red ware definitely appears [13], as well as larger White Painted containers (Fig 46) [13, 88]. Of particular interest is a Black Slip jug with irregular fluting [13]. In this regard Sørensen remarks that the vessel type of the Black Slip jug decreases in number and quality during Gjerstad’s group III [71]. Together with the appearance of Black-on-Red, this might be a possible indication for a comparably later transitional period [13].

### The Egyptian pottery context

In general terms, the Early Iron Age at Sidon coincides with the late Ramesside period and the beginning of the Third Intermediate Period in Egypt. Not much is known about trading contacts between Egypt and the Levantine coast during this period. Our main source of information comes from one of Egypt´s greatest literary works, the Report of Wenamun (Pap. Pushkin 120). The story tells the journey of an emissary of the Amun temple of Karnak, who travels on behalf of an unnamed Egyptian king to the northern Levant to bring back timber for the construction of the great Amun barge [89]. In this text Tanis, Dor, Tyre, Sidon and Byblos, as well as a place called *Jsr* are mentioned. So far only Tel Dor (see below) and Sidon have provided archaeological remains along the Levantine coast that can support these contacts.

**Fig 47:**
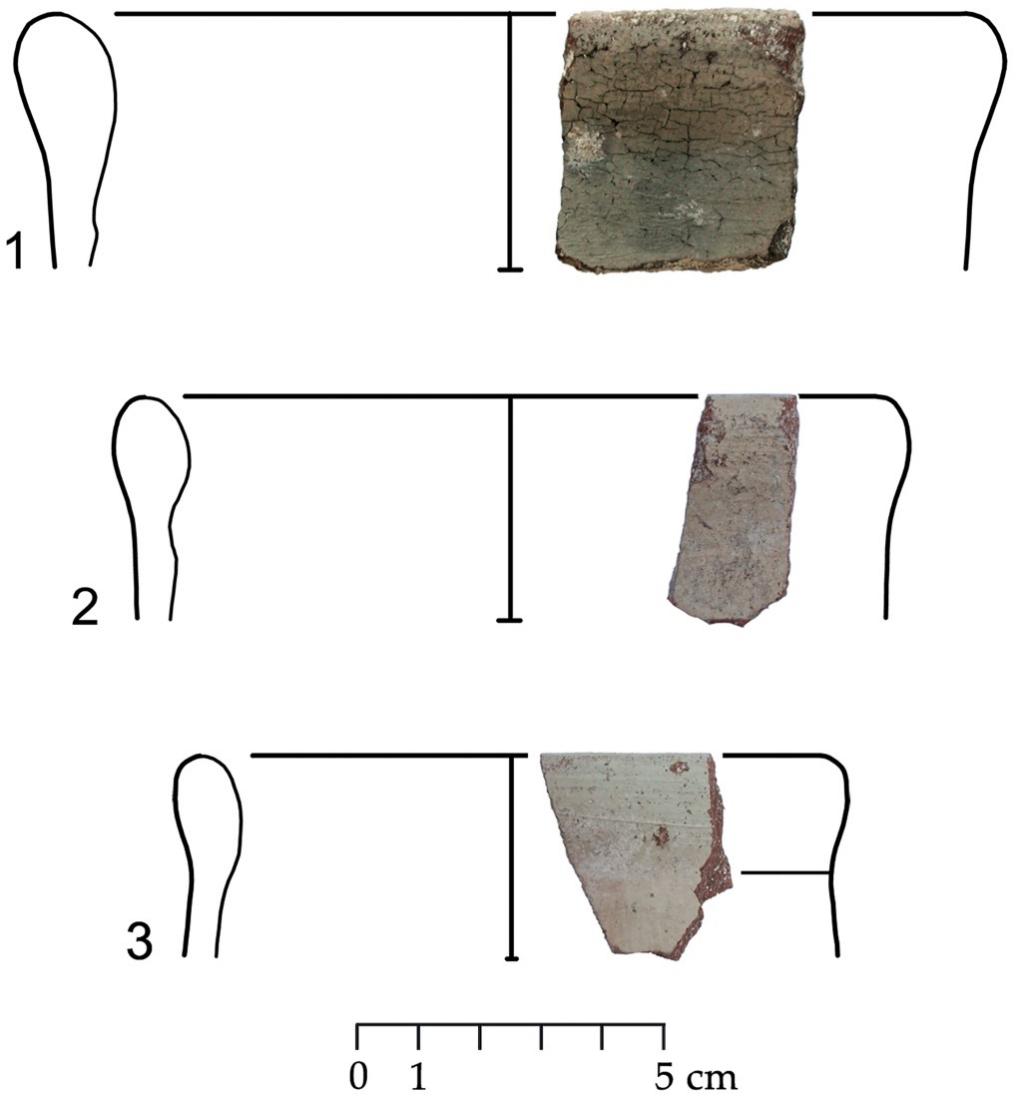
Amphora rims from Room 6: 1. S/125699/4510; 2. S/123892/4496; 3. S/120575/4446.

**Fig 48:**
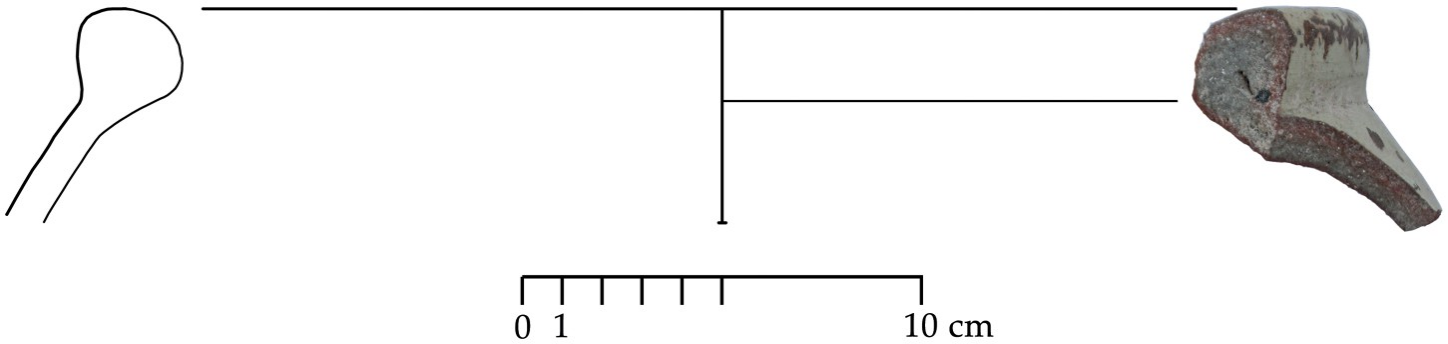
Rim of meat jar S/120631/4473 from Room 6.

**Fig 49:**
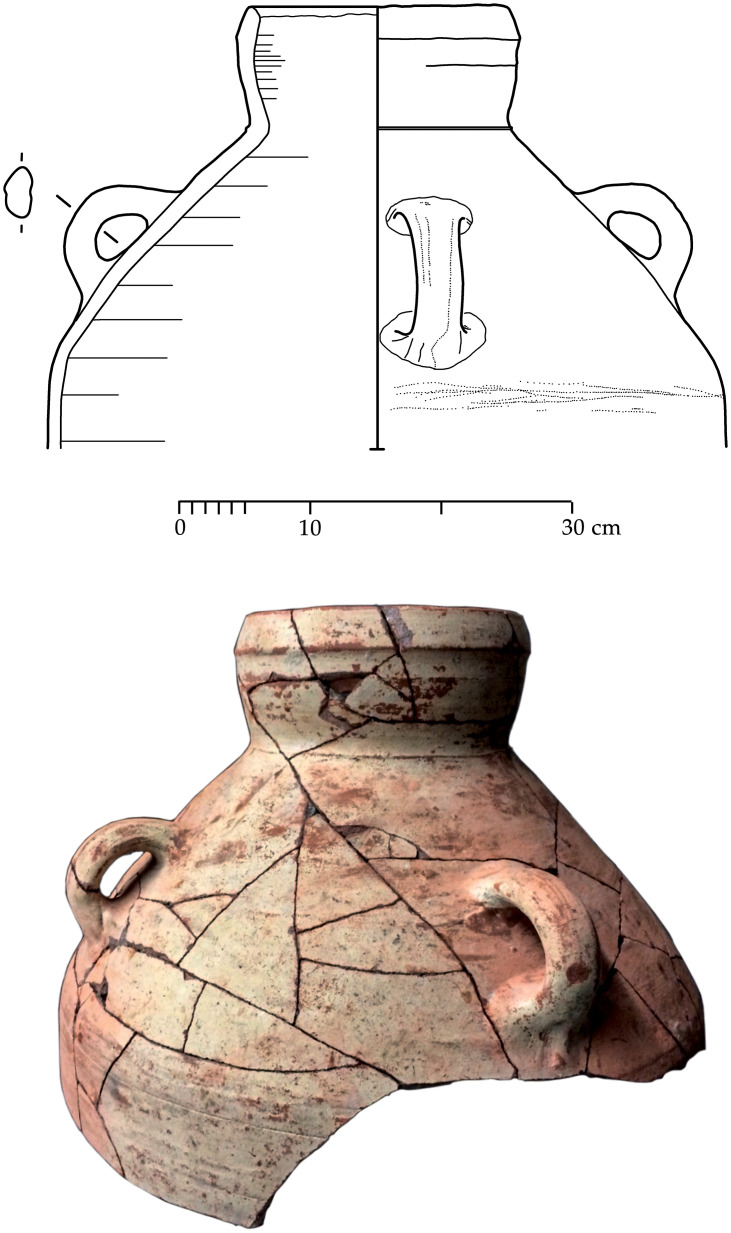
Large Storage jar with four handles, S/8098/8469, Nile G6a, white slip.

At Sidon only five pieces of Egyptian pottery were discovered in the Early Iron Age layers. Three are rim fragments of large amphoras (Phase B: S/125699/4510; Phase C: S/123892/4496; Phase E: S/120575/4446) and come from Room 6 (Fig 47). One is the rim fragment of a meat jar (S/120634/4473) (Fig 48), which was found in the same room in Phase D and the last piece is a large four-handled storage jar (S/8098/8469) coming from Room 5 of the same Phase (Fig 49).

Only tiny fractions of the rims of the amphoras and the meat jar were preserved. All these vessels were made of the Vienna System fabric Marl D [90]. According to Janine Bourriau and Paul T. Nicholson, the deposits for this clay could be in the Memphite region or in the Nile Delta [91], since the distribution of vessels made of this fabric is mainly in the north of Egypt. It seems that this fabric appears first during the reign of Thutmosis III [92] in the eastern Nile delta and was used during the later 18^th^ and in the 19^th^ Dynasties mainly for closed vessels. With the late 19^th^ and early 20^th^ Dynasties this fabric slowly vanishes and vessels that used to be made of Marl D fabric are now produced in Nile and Mixed clays [93].

The production of large Marl D amphoras in Egypt started according to David Aston [93] in the period between Amenhotep II and III and developed to a shape with a swollen longer neck, a melon-shaped body and a round bottom in the 20^th^ dynasty. This shape continues into the Third Intermediate Period [94], where it is then made of different clays.

The same holds true for the meat jars. These large storage vessels were thrown on the slow wheel in two parts, and then joined together creating a biconical body shape. Although dockets on vessels from Amarna indicated meat as their contents (hence the name ‘meat jars’), they were probably used to store all kind of commodities. These large storage vessels were produced from the late 18^th^ Dynasty [95] until the Third Intermediate Period [94]. Originally, without handles they seemed to have been equipped with a pair from the 20^th^ dynasty onwards [96]. But, like the amphoras, the original Marl D fabric got substituted during the 20^th^ Dynasty by other fabrics. Thus, it seems very likely that the rim fragments of both, amphoras and meat jar, are residual and were relocated by human activities inside Room 6 from deeper layers to those of the Iron Ages.

This was most likely not the case for the large four-handled storage jar found in Room 5, which was made of a different fabric than the above described pieces (Fig 49). It was made of the Nile clay fabric G6a, which is known since the 19^th^ Dynasty from Memphis [97] and which becomes popular during the late New Kingdom and in the Third Intermediate Period, especially for the production of large storage vessels [97]. Unfortunately, only the upper part of this jar was preserved. It has a wide rim opening which was trimmed inside with a tool. At the junction where the slightly swollen neck and the shoulder were joined sits a small decorative ridge to cover up this fact. The body was coiled in two parts, which were then joined at the base of the shoulder, creating a carination. Traces of trimming the excess clay are still visible at the joint. Four evenly spaced vertical handles sit on the lower shoulder just above the carination. An additional whitish slip covers the outside of the jar and the inside of its rim. Not burnished, it was fired to a whitish to pink colour. The hardness of the pottery suggests high firing temperatures. This should, in combination with the slip, increase the vessel´s density and possibly prevent rapid evaporation of liquids. Like many other storage vessels, it possibly was used to store all kinds of goods. At Sidon the jar was probably used as a large water container. It was found sunken into a small plastered platform with raised edges. These very likely served to collect some of the water that diffused through the vessel´s wall, cooling the jars content during this process. Close by a small scoop made of alabaster was found, which seemed to be connected to the use of this jar.

Jars like the Sidon piece are very rare and were found only in a few places. The closest parallel comes from a cemetery in Tell el-Yahudiyeh in the north-eastern Nile delta [98]. The cemetery consists of eight large tumuli, each containing several burials. Amongst the burials in tumulus VIII was a large storage jar with two handles, which was used as the container for a child burial. From the burials of the other seven tumuli came two scarabs, one of Ramses III and one of Ramses VI, indicating that this cemetery was in use during the 20^th^ Dynasty. From the area of the Royal horse studs in area Q/IV in Qantir a piece with a similar rim and two handles, but without the shoulder carination was unearthed in Stratum Bb [96] which is dated by the excavators to the very end of the 19^th^ and the early 20^th^ Dynasties (it is possible that due to the lack of the carination this piece represents an early version of this storage jar type) [96]. At Tanis predating the reign of Psusennes I, a similar jar was found in a cemetery that possibly belonged to the earliest settlers at the site and which was discovered under the enclosure wall of this king [99, 100]. It was used as a container for a child burial. Unfortunately, only the upper part of this vessel with three evenly spaced handles was preserved. Although the jar´s body was narrower than the one from the Sidon piece, it also had a carination at the shoulder. Based on archaeological and recently confirmed astronomical evidences this cemetery dates possibly into the early 21^st^ Dynasty. In a recent study the orientation of the large enclosing wall of Psusennes I was examined from the perspective of the astronomical conditions that prevailed at the time of its construction [101] and its possible orientation based on these. The results confirm Kitchen´s absolute dating of the 1^st^ regnal year of Psusennes I in 1039 BC [102] which in turn dated the cemetery prior to this date. According to the Report of Wenmun historians reconstructed that this city was founded either by the last ruler of the 20^th^ Dynasty, Ramses XI, or more likely the first ruler of the 21^st^ Dynasty, Smendes [103].

Two similar jars with only two instead of four handles were found at Tel Dor [39], where they were dated by the excavators to the phase late Iron Age IA, which might probably fall into the second half of the 11^th^ century BC [39]. Both the Tanis example and the pieces from Tel Dor were made of Nile clay.

Although, not many examples of this type of storage jar were found in Egypt, the few that have been discovered allow us to date the production of this large vessel between the reign of Ramses III and the early 21^st^ Dynasty. In absolute chronological terms this would be between 1175 and 1039 BC [104], which would fit with the ^14^C-dates proposed in this paper for Phase D.

The original use of these large storage vessels is of course no longer comprehensible, but when reading the report of Wenamun it is noticeable that by the end of the New Kingdom and the beginning of the Third Intermediate Period Egypt had to fall back on its agricultural products and goods that were manufactured as exchangeable commodities, simply due to a lack of the more precious metals as exchange commodities. However, it appears that throughout history agricultural products were most likely the main export commodities Egypt had to offer anyway. Since the storage jar was possibly made in the Nile delta, it is highly likely that the goods that were transported within it were also products from that geographical region [105].

## Results of the archaeological and analytical studies

In order to establish a secure Mediterranean chronology, the first requirement is reliable correlation of regional pottery styles, but which is seriously hampered by the diversity and wide geographic origin of the ceramic materials and the general lack of contextualized finds.

Directly following from its geographic position as major communication centre for long-distance trading and cultural exchange during the Late Bronze and Early Iron Age, today the Phoenician metropolis of Sidon provides us with the opportunity for archaeological research that is directed at its own interregional history. This Phoenician site has yielded, in stratified architectural sequence next to local Phoenician wares, some of the largest presently known assemblages of Greek and Cypriot pottery, as well as a wide variety of ceramic and other finds from Egypt, both in parallel with a sequence of short-lived organic samples. The now available sequence of ^14^C-dated architectural units and associated pottery finds at Sidon allows some reconsiderations about the synchronisation of regional ceramic styles. We emphasise that, due to the sheer amount of finds, it was possible to selectively exclude pottery finds that come from stratigraphically less well-defined contexts.

The first objective of the present study is to present the new archaeological and ^14^C-radiometric data from Sidon in a comparative manner, along with re-analysis of the data from other sites, such that it becomes more widely usable for the correlation and absolute dating of regional pottery styles between the Aegean and the eastern Mediterranean. This wider usability is a requirement for the construction of a supra-regional absolute chronological system. Our second objective is to furnish the new archaeological data with a re-analysis of certain aspects of the ^14^C-dating method that are now widely acknowledged as having critical importance for the construction of archaeological chronologies at high dating resolution, especially in the Levante, and that is the potential impact of local and regional variability in atmospheric ^14^C-reservoirs, of similarly small (annual-decadel) differences in ^14^C-laboratory intercalibration procedures, as well as of the many still unknown (unmeasured) fine-structures in the shape of the tree-ring calibration curve. The consistency of the AMS-dates and their stratigraphic sequence at Sidon speaks for the general reliability of the combined ^14^C-scientific and archaeological evidence. We note, in particular, the high consistency of our model-based ^14^C-redating of Megiddo, Tel Rehov, and Tel Tayinat with the previously published chronologies. This comparison is all the more satisfactory, since the results (purposely utilising the same data and models) were achieved using two quite different analytical approaches (Bayesian Sequencing, Gaussian Monte Carlo Wiggle Matching) and corresponding software (OxCal, CalPal). The precision of the ^14^C-analysis was contrasted, whenever possible, with the independently undertaken Phoenician, Greek, Cypriot and Egyptian pottery dating, but significant differences were not found.

Our dating project focuses on the examination of the archaeologically best-defined settlement phases at Sidon, beginning with Phase C. The available ^14^C-data does not allow any more refined definition of the two earlier Phases, namely A and B. Sidon Phase A, which together with the occupation layers underneath the temple, probably dates to sometime in the 13^th^ century BC, is one of the initial phases for use and deposition of pottery of Mycenaean type although the same pottery partly also appears in later phases of the Iron Age I period, hence presumably as residual material due to the extensive reworking of the architectural space. Equally conjectural are also both the beginning and duration of Phase B. This and the ensuing Phase C (1144–1074 calBC) seem to share the same chronological horizon that is culturally defined as the Protogeometric period in the Aegean. This is a period with restricted pottery exchange between the Aegean and the eastern Mediterranean, thus explaining the paucity of Greek ceramic finds in the layers of Phases B and C. Nevertheless, certain ceramic finds from Sidon such as a sherd from a pyxis and few plates, may date to the end of the Protogeometric period. The subsequent Phase D (1074–1002 calBC) correlates with the Aegean Early Geometric period dated by means of a bowl decorated with zigzag lines. The best dated–by means of AMS-dates–phases at Sidon are C and D that predate some important changes in the local cultural material. These innovations can be taken as marks of the chronological synchronisation between Sidon and other sites in the eastern Mediterranean. On the one hand, bichrome Phoenician pottery is represented for the first time with a few sherds in Sidon’s Phase E that we have now dated to the first half of the 10^th^ century. Interestingly, the bichrome Phoenician pottery appeared at other sites during the late Iron Age I that are independently dated more or less to the same period [106]. Differentiations in the local pottery production on the Levantine coast is due to regional variabilities in ceramic production whilst the inconsistency in the dating of the Greek pottery finds among eastern Mediterranean sites may be ascribed to flawed ceramic synchronisations and type definitions. This is at least what the recent data from the north Aegean at Sindos imply [6]. This previous data now finds independent confirmation in the new analytical data from Sidon. Even if the archaeological and analytical evidence from Sidon’s earlier Phases C to E are not considered, the fact remains that there is not a single carbon sample–among altogether 37 analysed olive pits and animal bones from Phases A to E and Phase I (we have only two outliers from Phases J and K)–that dates to after 900 calBC. At least four sherds, including two pendent semicircle skyphoi of type 4 and 5 and two monochrome bowls, all of which date well into the Middle Geometric II, come from well-stratified contexts of Phase I that dates prior to 900 calBC. Additionally, there are dozens of other pendent semicircle plates and skyphoi of type 4 and 5 –although found in less well-defined contexts but almost certainly associated with Phase I or earlier phases–that cannot be dated to after 900 calBC either.

## Conclusions

The new data from Sidon provide support for the high Aegean chronology, in the present case specifically by raising the Early and Middle Geometric period to the 11^th^ and 10^th^ centuries BC. The results of this study are thus consistent with the chronological data recently obtained in the northern Aegean [4, 6], at Phoenician sites in southern Spain [7, 107], in northern Africa [1, 5] as well as with recent suggestions about a higher Mediterranean chronology deriving evidence from holistic examination of available data in the central and western Mediterranean [108]. This suggests not only a much longer time span for the Geometric period in the Aegean but also a different periodisation pattern. The implications of the revised Aegean chronology on what has been called Mediterranean chronology and especially the Iron Age chronology of the Levant must now be reassessed not only for the wider regional ceramic synchronisms between the Aegean and the eastern Mediterranean, but also among several microregions along the Levantine coast.
